# Dietary fibre directs microbial tryptophan metabolism via metabolic interactions in the gut microbiota

**DOI:** 10.1038/s41564-024-01737-3

**Published:** 2024-06-25

**Authors:** Anurag K. Sinha, Martin F. Laursen, Julius E. Brinck, Morten L. Rybtke, Anna Pii Hjørne, Nicola Procházková, Mikael Pedersen, Henrik M. Roager, Tine R. Licht

**Affiliations:** 1 National Food Institute, Technical University of Denmark, Kongens Lyngby, Denmark; 2 Department of Nutrition, Exercise and Sports, University of Copenhagen, Copenhagen, Denmark

**Keywords:** Microbiome, Transcription, Microbial ecology, Bacteriology

## Abstract

Tryptophan is catabolized by gut microorganisms resulting in a wide range of metabolites implicated in both beneficial and adverse host effects. How gut microbial tryptophan metabolism is directed towards indole, associated with chronic kidney disease, or towards protective indolelactic acid (ILA) and indolepropionic acid (IPA) is unclear. Here we used in vitro culturing and animal experiments to assess gut microbial competition for tryptophan and the resulting metabolites in a controlled three-species defined community and in complex undefined human faecal communities. The generation of specific tryptophan-derived metabolites was not predominantly determined by the abundance of tryptophan-metabolizing bacteria, but rather by substrate-dependent regulation of specific metabolic pathways. Indole-producing *Escherichia coli* and ILA- and IPA-producing *Clostridium sporogenes* competed for tryptophan within the three-species community in vitro and in vivo. Importantly, fibre-degrading *Bacteroides thetaiotaomicron* affected this competition by cross-feeding monosaccharides to *E. coli*. This inhibited indole production through catabolite repression, thus making more tryptophan available to *C. sporogenes*, resulting in increased ILA and IPA production. The fibre-dependent reduction in indole was confirmed using human faecal cultures and faecal-microbiota-transplanted gnotobiotic mice. Our findings explain why consumption of fermentable fibres suppresses indole production but promotes the generation of other tryptophan metabolites associated with health benefits.

## Abstract (web summary)

Substrate and fibre availabilities determine whether the gut microbiota generates beneficial or detrimental tryptophan metabolites.

## Main

Tryptophan is an essential amino acid that is metabolized in the gastrointestinal tract by both host and gut microbiota, resulting in a variety of metabolites that can affect host metabolism and homeostasis^1,2^. Tryptophan is readily used by several gut microbial species that catabolize it to metabolites including indole, indolelactic acid (ILA), indoleacrylic acid (IAcrA), indolepropionic acid (IPA), indoleacetic acid (IAA), indolealdehyde (IAld), tryptamine and so on^1,3,4^. These metabolites regulate host biological processes such as maintenance of epithelial barrier integrity, immune response, protection against pathogens, inflammation and metabolic disorders^1,3–5^. Many of them elicit beneficial effects, while others may lead to adverse responses in the host^1^. For example, ILA, IAA and IAld have been shown to stimulate human CD4^+^ T cells to produce IL-22 and reprogramme intraepithelial CD4^+^ T helper cells^1,3,6^, thereby promoting tolerance against dietary antigens^7^. IPA regulates mucosal integrity through the Toll-like receptor signalling pathway^8,9^, and is negatively correlated with type 2 diabetes^10,11^, regulates gut permeability^12^, inhibits atherosclerosis^13^, and has antioxidant, anti-inflammatory and neuroprotective properties^14–16^. By contrast, indole produced in the gut is converted into the uraemic toxin indoxyl sulfate (IS) in the liver and accumulates in patients with chronic kidney disease (CKD), contributing to the pathophysiology of the disease^1,17–19^. In addition, high indole concentrations in the colon are reported to promote persistent infection with *Clostridium difficile*^20^.

**Fig. 1:**
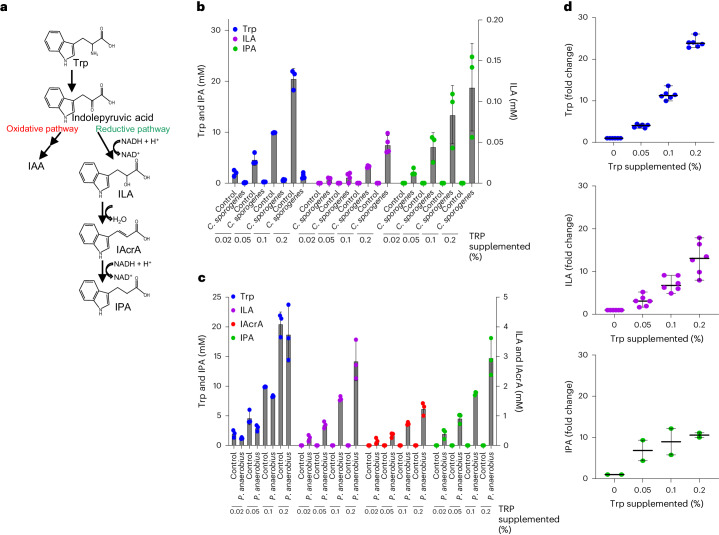
Tryptophan supplementation increases tryptophan-derived Stickland fermentation products. **a**, Schematic representation of the Stickland fermentation pathway. Stickland fermentation of Trp generates either the oxidative pathway product IAA or the reductive pathway products ILA, IAcrA and IPA^12^. **b**, Concentrations of Trp, ILA and IPA in mGAM (control) and culture supernatants of *C. sporogenes* grown in mGAM supplemented with final concentrations of 0.02%, 0.05%, 0.1% and 0.2% free tryptophan. Bars and error lines indicate the mean ± s.d. of three to four independent biological replicates. **c**, Concentrations of Trp, ILA, IAcrA and IPA in mGAM (control) and culture supernatants of *P. anaerobius* grown in mGAM supplemented with final concentrations of 0.02%, 0.05%, 0.1% and 0.2% free tryptophan. Bars and error lines indicate the mean ± s.d. of three independent biological replicates. **d**, Fold change in concentrations of tryptophan metabolites in the culture supernatants of six infant faecal communities cultured either in unsupplemented YCFA medium or in YCFA supplemented with 0.05% or 0.1% or 0.2% of free tryptophan. Specific metabolite concentrations are normalized to the basal level of the given metabolite in the growth medium without tryptophan supplementation. Lines and error lines indicate the mean ± s.d. IPA was detected only in two of the six infant faecal communities owing to the lack of producer species *P. anaerobius* in the four remaining faecal samples (Extended Data Fig. 2d). Absolute values of metabolites in the individual faecal cultures and the microbiota compositions are shown in Extended Data Fig. 2. Source data

Indole is the most abundant tryptophan metabolite detected in mouse caecal contents as well as in human faeces, contributing to 50–75% of the total tryptophan metabolites and reaching concentrations up to 2.6 mM (refs. ^21,22^). Intestinal indole is produced by *Escherichia coli*, *Bacteroides* spp. and a number of other gut species through a single enzymatic process catalysed by the *tnaA*-encoded tryptophanase enzyme, which hydrolyses tryptophan into indole, pyruvate and ammonia^23–26^. Another metabolic pathway, Stickland fermentation, was first described in *Clostridium sporogenes* and converts tryptophan into the oxidative pathway product IAA, and the reductive pathway products ILA, IAcrA and IPA (Fig. 1a)^12,27,28^. Stickland fermentation is thus a coupled chemical reaction in which one amino acid gets oxidized, while another amino acid gets reduced^27^. *C. sporogenes* obtain their energy primarily through Stickland fermentation of amino acids in which oxidative metabolism of one amino acid generates ATP via substrate-level phosphorylation, while the redox balance is maintained by reducing another amino acid^12,27–29^. In addition, many *Bifidobacterium* and Lactobacillaceae species are known to produce ILA from tryptophan in the gut, catalysed by a specific aromatic lactate dehydrogenase (Aldh) enzyme^3,7,30,31^. The diverse range of tryptophan metabolites produced by the intestinal multispecies community^1^ suggests that bacterial competition for available tryptophan may drive their accumulation in the gut. However, despite their important roles in host homeostasis, the factors regulating the generation of these metabolites in the gut remain unknown. A large number of studies indicate that fibre intake reduces microbial catabolism of protein^32–35^. Similarly, fibre intake is well known to reduce intestinal transit time^36^, which prevents the depletion of carbohydrates available to microorganisms in the large intestine and thereby reduces the generation of harmful products resulting from microbial decomposition of dietary or host-derived proteins^37^. Notably, recent studies additionally suggest that consumption of fermentable fibre somehow affects microbial tryptophan metabolism. One epidemiological study of five very diverse cohorts reveals that higher daily fibre intake is strongly associated with higher serum levels of IPA and lower levels of IS^10^. Conversely, IS is positively associated with total protein and specifically tryptophan intake^10^. A positive correlation between serum concentrations of IPA and daily dietary fibre intake is identified in studies of a Finnish population^11,38^ and in a UK cohort^39^. Furthermore, a recent meta-analysis concludes that serum IS correlates negatively with dietary fibre intake in individuals with CKD^40^, and intervention with dietary fibre significantly reduces serum IS in patients undergoing haemodialysis^41^. In addition, interindividual variations in IS are recently reported not to be affected by a homogenous diet low in fermentable fibres^42^.

Therefore, we set out to unravel how production of specific tryptophan metabolites by the intestinal community is affected by the availability of the substrate as well as by the presence of fermentable carbohydrates in the gut environment. We show that tryptophan availability, degradation of fermentable carbohydrates and the presence of specific bacterial species govern the balance between tryptophan metabolites formed by Stickland versus tryptophanase pathways in vitro and in vivo. Our results provide key mechanisms explaining observations from multiple human studies reporting associations between fibre intake and microbiome metabolic output.

## Results

### Substrate availability governs the production of tryptophan metabolites

**Extended Data Fig. 1:**
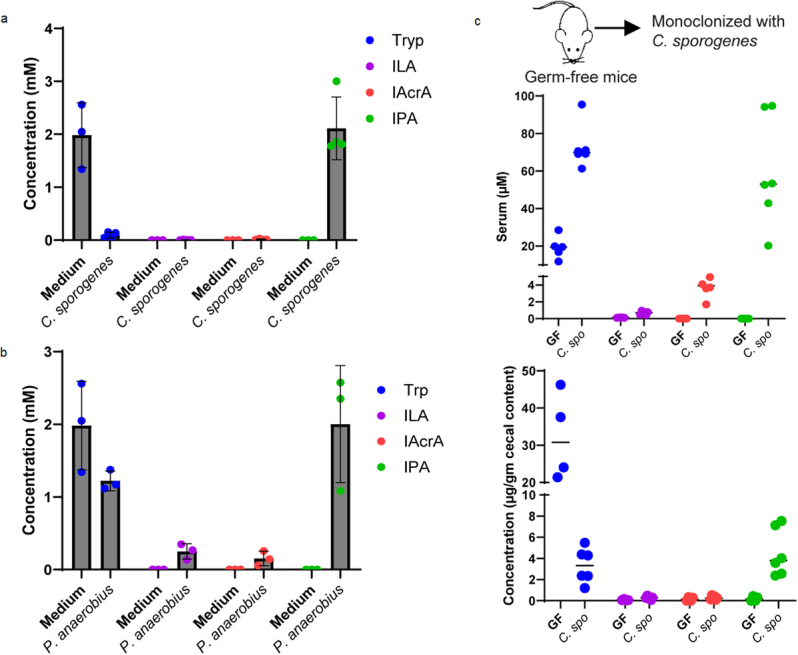
The gut bacteria *Clostridium sporogenes* and *Peptostreptococcus anaerobius* produce tryptophan metabolites. (**a**) Tryptophan metabolites in the medium and culture supernatants of *C. sporogenes* grown for 48 hrs in mGAM medium. Bars and error lines show mean ± s.d. of four independent biological replicates for *C. sporogenes* and three for control medium. (**b**) Tryptophan metabolites in the medium and culture supernatants of *P. anaerobius* grown for 48 hrs in mGAM medium in triplicates. Bars and error lines show mean ± s.d. of three independent biological replicates. (**c**) Tryptophan metabolites in serum (five) and in cecum (four) of germ-free (GF) and six gnotobiotic mice monocolonized with *C. sporogenes* (*C. spo*). Source data

**Extended Data Fig. 2:**
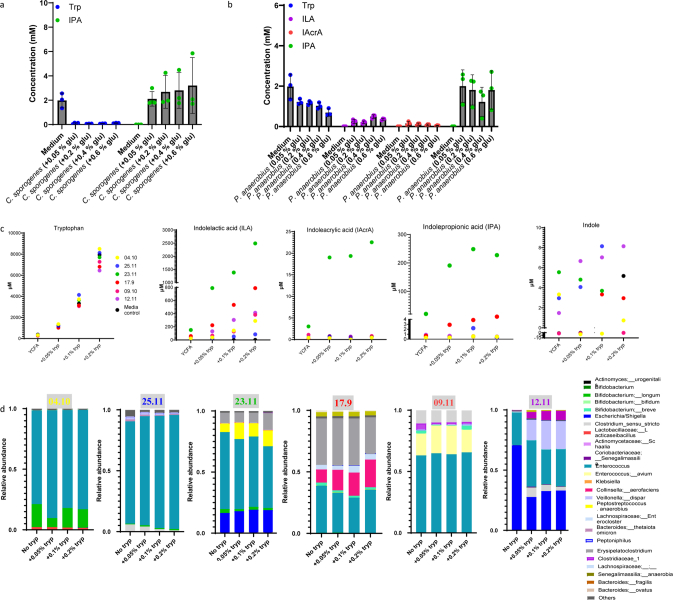
Effect of glucose and tryptophan supplementation on production of tryptophan metabolites. (**a**) Concentration of tryptophan metabolites in the mGAM medium and culture supernatants of *C. sporogenes* grown in mGAM medium supplemented with final concentrations of 0.05, 0.2 or 0.4 and 0.6 % glucose. Bars and error lines show mean ± s.d. of three independent biological replicates. (**b**) Concentration of tryptophan metabolites in the mGAM medium and culture supernatants of *P. anaerobius* grown in mGAM medium supplemented with final concentrations of 0.05, 0.2 or 0.4 and 0.6 % glucose. Bars and error lines show mean ± s.d. of three independent biological replicates. (**c**) Absolute concentrations of tryptophan metabolites in the culture supernatants of six infant faecal microbiotas and culture medium control (media control). The six different infant faecal microbial communities are cultured either in YCFA medium or YCFA supplemented with 0.05 or 0.1 or 0.2 % of free tryptophan. (**d**) 16S rRNA gene sequencing profile of the six infant faecal microbiota compositions. Source data

To study gut microbial tryptophan catabolism, we used two model species known to perform Stickland fermentation^12,27^ (Fig. 1a). We confirmed in vitro that *C. sporogenes* and *Peptostreptococcus anaerobius* produced the specific tryptophan metabolites, ILA, IAcrA and IPA, resulting from the reductive pathway of the Stickland fermentation, while tryptophan was consumed (Extended Data Fig. 1a,b). In contrast to germ-free (GF) mice, mice mono-colonized with *C. sporogenes* contained the tryptophan metabolites in the caecum and serum (Extended Data Fig. 1c), confirming the strict microbial origin of these metabolites^43^. In addition, caecal concentrations of tryptophan were significantly reduced in *C. sporogenes*-colonized mice compared with GF mice. Furthermore, as tryptophan was almost completely consumed by *C. sporogenes* in vitro (Extended Data Fig. 1a), we hypothesized that levels of Stickland fermentation products would depend upon the availability of this substrate. Indeed, we observed a clear dose-dependent accumulation of ILA and IPA in the culture supernatant of both *C. sporogenes* and *P. anaerobius* upon tryptophan supplementation in the medium (Fig. 1b,c), implying that tryptophan availability drives the production of ILA and IPA. Next, we assessed whether higher carbohydrate availability affected production of ILA and IPA, considering that *C. sporogenes* does ferment carbohydrates, although they are not essential for growth of this species if amino acids are present in the environment^27^. No significant change in the tryptophan metabolites was observed upon supplementation of five- to tenfold higher glucose in the growth medium (Extended Data Fig. 2a,b), suggesting that Stickland fermentation is unaffected by the presence of carbohydrates in the environment.

Next, we investigated the effects of substrate availability on tryptophan metabolite production in the relatively simple gut microbial community of infants. Six infant faecal samples from a previous study^3^ were selected based on the presence of ILA-producing *Bifidobacterium* species^3^ or ILA- and IPA-producing *P. anaerobius*^12^. In agreement with the monoculture experiments, tryptophan supplementation significantly increased ILA and IPA production in the infant microbiota communities (Fig. 1d and Extended Data Fig. 2c). In addition, 16S rRNA amplicon sequencing revealed that the different tryptophan supplementations did not lead to noteworthy differences in the individual community composition, suggesting that the increase in ILA and IPA was driven by higher substrate availability and not attributed to a change in the abundance of specific producer species (Extended Data Fig. 2d).

### Carbohydrate availability affects microbial tryptophan metabolism

**Fig. 2:**
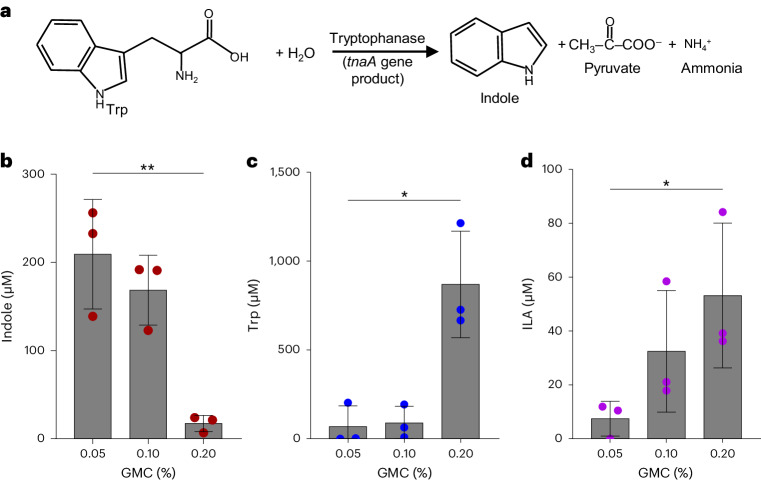
Carbohydrate supplementation inhibits indole production in faecal cultures. **a**, Schematic representation of tryptophanase-mediated catabolism of tryptophan to produce indole, pyruvate and ammonia. **b**–**d**, Concentrations of indole (**b**), Trp (**c**) and ILA (**d**) in the faecal culture supernatants after cultivation in YCFA medium supplemented with 0.05%, 0.1% or 0.2% glucose, maltose and cellobiose, collectively referred to as GMC. One infant faecal sample (23.11) was selected for cultivation in three replicates as it contained both ILA and IPA producers (for example, *P. anaerobius* and *Bifidobacterium longum*) and indole producers (*Escherichia*). Metabolites were normalized to the final OD_600_ of the culture in the individual culture supernatants. Results are the mean ± s.d. of three independent experiments. Statistical analysis was done using a two-tailed unpaired *t*-test comparing lowest and highest GMC concentrations, with *P < 0.05; **P < 0.01 (indole, *P* = 0.0061; Trp, *P* = 0.0126; ILA, *P* = 0.0456). Individual replicates and their 16S rRNA profiles are shown in Extended Data Fig. 3. Source data

**Extended Data Fig. 3:**
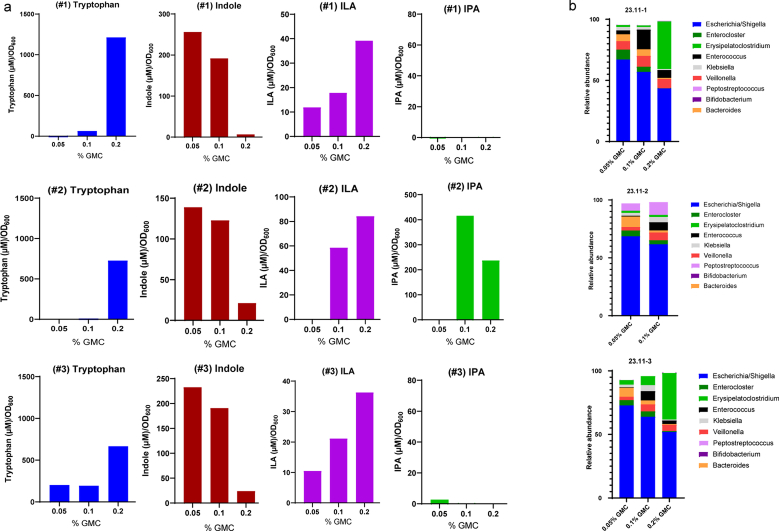
Carbohydrates supplementation inhibits indole production and stimulates ILA production in faecal cultures. (**a**) Normalized concentrations of tryptophan metabolites in the culture supernatants from faecal cultures in YCFA medium supplemented either with 0.05 or 0.1 or 0.2 % each of glucose (G), maltose (M) and cellobiose (C), collectively referred here as GMC. A single infant faecal sample (23.11) was selected for cultivation in three replicates since it contained both ILA/IPA producers (for example *P. anaerobius* and *B. longum*), and indole producers (*Escherichia*). Metabolites in the individual culture supernatants were normalized against final OD_600_ of the culture. All individual replicates are shown here. (**b**) 16S rRNA profiles of all replicates. Note that *Peptostreptococcus* is frequently lost thus preventing IPA accumulation in many samples (for example replicate #1 and #3). 16S rRNA profile of 0.2 % of #2 replicate is missing due to the loss of the sample during processing. Source data

While ILA, IAcrA and IPA are generated by a multistep reductive Stickland fermentation (Clostridiales species) or, in the case of ILA, also by a two-step transamination and reduction (lactic acid bacteria and *Bifidobacterium* species), these pathways are encoded by only a few specific members of the human gut microbiota. By contrast, indole is produced from tryptophan in a single catabolic step by a multitude of gut bacteria encoding the tryptophanase enzyme gene *tnaA* (Fig. 2a)^23–26,44^. In *E. coli*, which is one of the main indole producers in patients with CKD^23^, the *tnaA* gene is under the control of carbon catabolite repression and its expression is thus inhibited by the presence of simple carbohydrates such as glucose, arabinose and pyruvate^44,45^. We therefore hypothesized that addition of simple carbohydrates to the growth medium would inhibit indole production in the faecal culture. To test this, we cultured an infant faecal sample, confirmed by 16S rRNA amplicon sequencing to contain *Escherichia* species, in yeast casitone fatty acids (YCFA) medium with low to high concentrations of glucose, maltose and cellobiose as per protocol^46^ (Fig. 2b). Indeed, at low carbohydrate concentrations, indole was readily produced while at higher concentrations, its production was almost completely inhibited, confirming our hypothesis (Fig. 2b and Extended Data Fig. 3a). In addition, 16S rRNA amplicon sequencing confirmed that a high abundance of *Escherichia* was maintained even after higher carbohydrate supplementation (Extended Data Fig. 3b). Thus, we conclude that supplementation of carbohydrates inhibited indole production in the complex community without altering the abundance of the producing species. Importantly, inhibition of indole production concomitantly caused more tryptophan (substrate) to remain available in the supernatants from cultures supplemented with 0.2% carbohydrates (Fig. 2c and Extended Data Fig. 3a). Higher tryptophan availability led to increased production of ILA (Fig. 2b) in line with the monoculture and faecal culture experiments (Fig. 1). Conversely, supplementation with limited amounts (0.05%) of carbohydrates resulted in more tryptophan being converted into indole and a reduction in ILA production (Fig. 2d). Thus, we observed clear inverse associations between indole and ILA accumulation. The supplementation experiments in complex communities confirm that microorganisms compete for available tryptophan to produce either indole or other gut microbial tryptophan products such as ILA, and that the outcome of this competition is influenced by the availability of carbohydrates in the environment.

### Pectin inhibits indole production through cross-feeding

**Fig. 3:**
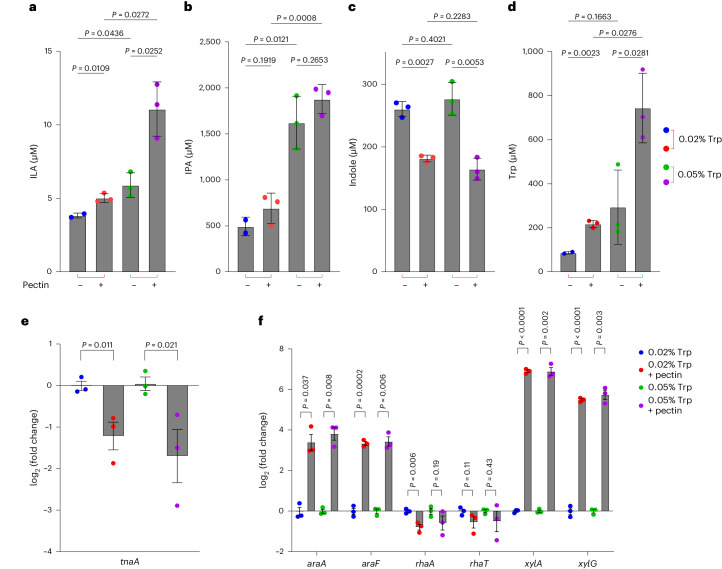
Tryptophan and fibre supplementation alters production of tryptophan metabolites in a defined community of gut microorganisms in vitro*.* Tryptophan metabolites produced by the defined community in vitro. *E. coli* was selected as the major indole producer, *B. thetaiotaomicron* was selected as the fibre degrader and *C. sporogenes* was selected because of its ability to generate Stickland fermentation products. **a**–**d**, Concentrations of ILA (**a**), IPA (**b**), indole (**c**) and Trp (**d**) in the supernatants of the defined community cultured in mGAM supplemented with either 0.02% or 0.05% free tryptophan and with or without 0.5% apple pectin. Bars and error lines indicate the mean ± s.d. of three independent biological replicates. Statistical analysis was done using the Brown–Forsythe ANOVA test using an unpaired two-tailed *t*-test with Welch’s correction. Only two replicates are shown in the group of 0.02% Trp without pectin for ILA, IPA and Trp owing to technical issues during analysis. **e**, Relative expression from RT-qPCR targeting *tnaA* mRNA in *E. coli* in response to tryptophan and pectin supplementation. **f**, Relative expression from RT-qPCR targeting mRNAs of arabinose-utilizing genes (*araA* and *araF*), rhamnose-utilizing genes (*rhaA* and *rhaT*) and xylose-utilizing genes (*xylA* and *xylG*) in *E. coli* in response to tryptophan and pectin supplementation. Total RNA was extracted from early stationary phase cultures (∼1 OD), and mRNA levels were measured as described in Methods and reported as relative difference (fold change) to the 0.02% Trp condition. Results are the mean ± s.e.m. of three independent experiments. Unpaired two-tailed *t*-tests were performed on the expression ratios to determine the statistical significance of the relative expression differences. *P* values are shown in the figure panels. Source data

**Extended Data Fig. 4:**
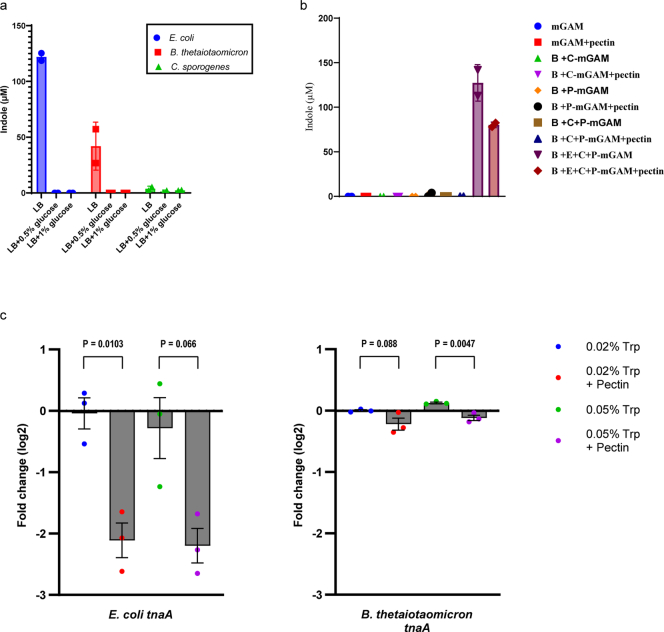
Supplementation with glucose or pectin inhibits indole production by repressing *tnaA* gene expression. (**a**) Indole production measured in the culture supernatants of *E. coli*, *B. theta* and *C. sporogenes* cultured individually in either LB broth or LB broth supplemented with 0.5 % glucose or 1 % glucose. Bars and error lines show mean ± SD of two independent biological replicates. (**b**) Indole production measured in the culture supernatants of defined communities consisting of *E. coli* (E), *B. theta* (B)*, P. anaerobius* (P) and *C. spo* (C) cultured in either mGAM or mGAM supplemented with 0.5 % pectin. Bars and error lines show mean ± SD of two independent biological replicates. (**c**) Defined community consists of *E. coli*, *B. theta* and *C. spo* are cultured together in mGAM medium supplemented with either 0.02 or 0.05 % free tryptophan and with or without 0.5 % apple pectin. Total RNA was extracted after 24 hrs fermentation and mRNA levels were measured as described in methods and reported as relative difference (fold change) to the 0.02% Trp condition. Bars and error lines show mean ± SEM of three independent biological replicates. Metabolites data are shown in Fig. 3. Relative expression from RT-qPCR to measure *tnaA* mRNA levels in *E. coli* and in *B. theta* in response to pectin supplemented (0.5%) in the growth medium after 24 hrs fermentation at either low (0.02%) or high tryptophan (0.05%) concentrations is shown here. Unpaired two-tailed t-tests were performed on the expression ratios to determine the statistical significance of the relative expression differences. P values are shown in the figure panels. Source data

Because simple sugars from the diet do not reach the colonic microorganisms, we addressed whether catabolism of complex fibres by gut microorganisms would cross-feed simple sugars that infer catabolite repression in *E. coli*, and thereby affect indole production. For this, we constructed a simple microbial community comprising the three model species: *E. coli* (indole producer), *Bacteroides thetaiotaomicron* (indole producer^24^, pectin degrader) and *C. sporogenes* (producer of Stickland fermentation products). Measurements of monoculture supernatants from *E. coli*, *B. thetaiotaomicron* and *C. sporogenes* grown in Luria Bertani broth revealed that indole production was almost four- to fivefold lower in *B. thetaiotaomicron* than in *E. coli*, and neglectable in *C. sporogenes* (Extended Data Fig. 4a). When these species, in addition to *P. anaerobius*, were co-cultured in different combinations in Gifu Anaerobic Medium, Modified (mGAM) broth, only the presence of *E. coli* resulted in indole accumulation in the culture supernatant (Extended Data Fig. 4b), revealing *E. coli* as the main indole producer in the defined community. The defined three-species community was then cultured in mGAM containing low (0.02%) and high (0.05%) amounts of tryptophan, with or without apple pectin supplementation. In agreement with observations from monocultures (Fig. 1), a 2.5-fold higher supplementation of tryptophan resulted in two- to threefold higher levels of ILA and IPA in the supernatants of the three-species community, confirming that substrate availability determines the Stickland fermentation (Fig. 3a,b). Furthermore, the presence of pectin in the growth media consistently inhibited indole production by 40–50% compared with when pectin was not added, both in the low- and high-tryptophan groups (Fig. 3c). By contrast, tryptophan and ILA both increased in the presence of pectin, and the same trend was observed for IPA (Fig. 3a,d). Pectin availability thus directed tryptophan metabolism towards less indole production and increased Stickland fermentation in the three-species system.

Monosaccharides such as arabinose, a constituent sugar of pectin, together with xylose and rhamnose, are known to repress *tnaA* gene expression in *E. coli*^44,45^. To test whether cross-feeding of monosaccharides resulting from *B. thetaiotaomicron* pectin degradation repressed *tnaA* gene expression in *E. coli*, we monitored messenger RNA abundance by reverse transcription quantitative PCR (RT-qPCR) from the early stationary phase cultures of the defined three-species community. Indeed, when the community was grown in presence of pectin, *E. coli tnaA* gene expression was inhibited two- to fourfold (Fig. 3e), explaining the inhibition of indole production and tryptophan consumption. Further inhibition of the *tnaA* gene in both *E. coli* and *B. thetaiotaomicron* was observed when samples were collected after 24 h of fermentation (Extended Data Fig. 4c). Interestingly, arabinose- and xylose-utilizing (but not rhamnose-utilizing) genes were upregulated by 16- to 64-fold, respectively, in *E. coli* in the presence of pectin, suggesting that uptake of these monosaccharides was increased in *E. coli* owing to cross-feeding with products of pectin degradation (Fig. 3f). This is largely in agreement with a previous study showing that *B. thetaiotaomicron* digests pectin, which upregulates arabinose-, xylose- and rhamnose-utilizing genes in *E. coli* when the two species are co-cultured^47^.

These findings show that pectin degradation results in cross-feeding of simple carbohydrates to *E. coli*, which, owing to catabolite repression, inhibits expression of *tnaA*. Consequently, the conversion of tryptophan into indole is inhibited, making more tryptophan available for *C. sporogenes* Stickland fermentation.

### Dietary pectin and tryptophan influence microbial metabolites in mice

**Fig. 4:**
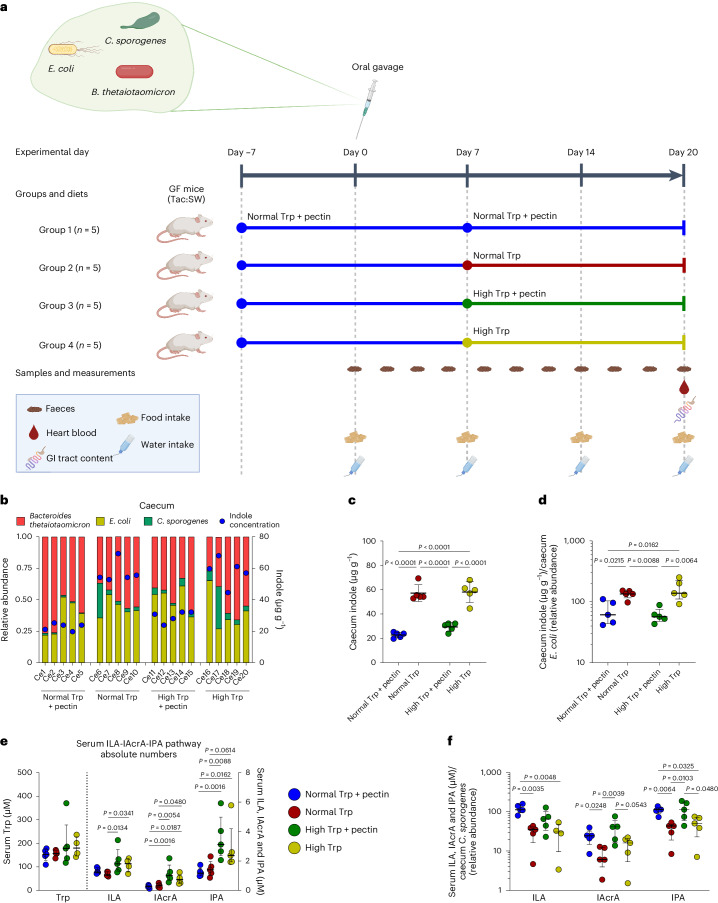
Dietary fibre and tryptophan supplementation modulates production of tryptophan metabolites by a defined community in vivo*.* **a**, Schematic representation of the experimental plan to evaluate the effect of dietary tryptophan and pectin on the production of tryptophan metabolites in vivo. GF mice were placed in four groups (*n* = 5 per group) and fed a diet containing 2 g kg^−1^ tryptophan and 50 g kg^−1^ pectin for 7 days for adaptation. They were then orally gavaged with a mixed culture of *E. coli*, *B. thetaiotaomicron* and *C. sporogenes* in equal amounts (OD_600_) and remained for another 7 days on the same diet for stabilization. The diets were then changed; the mice were fed a diet with either 2 g kg^−1^ or 16 g kg^−1^ tryptophan, with or without 50 g kg^−1^ pectin for two more weeks. Diet compositions are described in Supplementary Table 1. Samples were collected as shown in the scheme. **b**, The 16S rRNA gene sequencing profiles show the composition of the defined community in the caecum of each mouse in the four groups, overlaid with indole values measured in the individual caeca. **c**, Absolute concentrations of indole in caeca. **d**, Indole concentration in the caeca, normalized to the relative abundance of *E. coli*. **e**, Absolute concentrations of Trp, ILA, IAcrA and IPA in serum. **f**, Serum tryptophan metabolites (ILA, IAcrA and IPA) normalized to *C. sporogenes* relative abundance in caecum. For **c**, lines and error bars indicate means and standard deviations, respectively; for **d**–**f**, lines and error bars indicate medians and interquartile ranges (IQRs), respectively. Statistical analysis was done across groups within each metabolite measured using one-way ANOVA (**c**) or Kruskal–Wallis tests (**d**–**f**), using uncorrected Fisher’s LSD or Dunn’s post hoc tests (two tailed) to compare between individual groups. For **c**–**f**, *n* = 5 mice samples per group were used for statistical analysis. However, for **e** and **f**, one value for tryptophan and ILA was excluded as an extreme outlier (Grubbs test, alpha < 0.01). *P* values are shown in the figure panels. Panel **a** was created with BioRender.com. Source data

**Extended Data Fig. 5:**
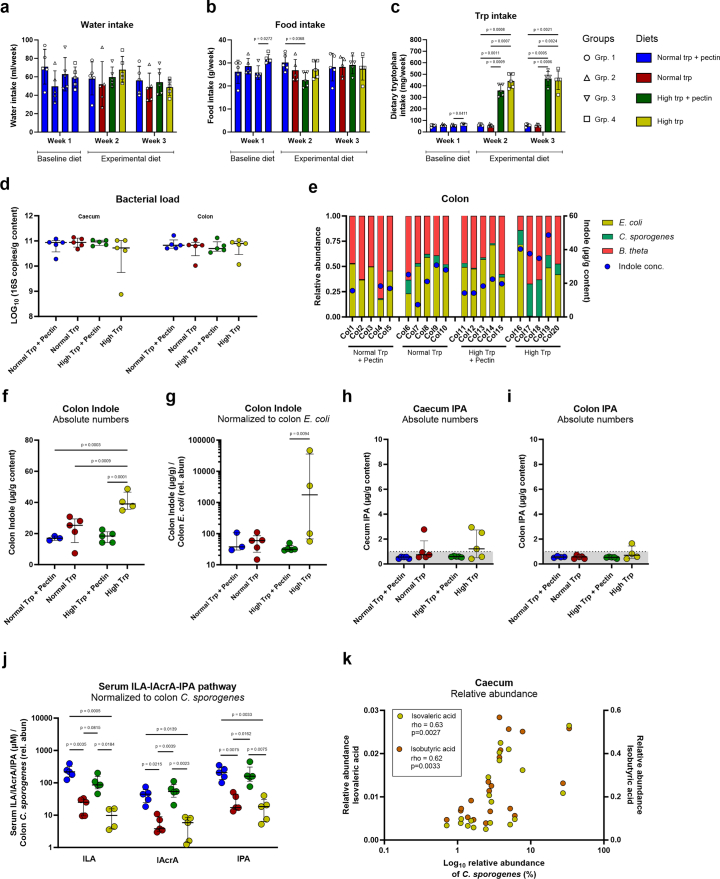
Additional data from the defined community experiment *in vivo* assessing the effect of dietary fibre and tryptophan supplementation on production of tryptophan metabolites. (**a**) Weekly water intake, (**b**) weekly food intake and (**c**) calculated weekly tryptophan intake from individual mice in the different experimental groups (n = 5 biologically independent animals per group) described in Fig. 4a. Bars and error bars indicate mean ± s.d. No significant differences were found between groups in water intake (p_group_overall_ = 0.61) or food intake (p_group_overall_ = 0.39) according to Two-way repeated measures ANOVA (p > 0.05 for all pairwise comparisons between feeding groups at week 2 and 3 after Bonferroni correction). Tryptophan intake differed significantly between groups (p_group_overall_ < 0.0001) and were significantly higher for group 3 and 4 compared to group 1 and 2 in both week 2 and 3 (p < 0.01 for all pairwise comparisons across, but not within, trp feeding groups after Bonferroni correction). (**d**) Total bacterial load quantified using qPCR, showing median and error bars showing IQR. No significant differences were found using Kruskal-Wallis tests (n = 5 biologically independent animals per group). (**e**) 16S rRNA gene sequencing profiles show the composition of the defined community in colon of each individual mouse, with indole values in the colon overlaid. Indole values are missing for few mice due to the extremely low samples amounts present in their colon. (**f**) Absolute indole concentration in the colon. (**g**) Indole concentration in the colon, normalized to the relative abundance of *E. coli* in colon. (**h, i**) Absolute abundance of IPA in (h) cecum and in (i) colon, with grey shaded area indicating background noise. (**j**) Serum tryptophan metabolites normalized to *C. sporogenes* relative abundance in colon. (**k**) Scatter plot and Spearman’s rank correlation of relative abundances of isovaleric acid and isobutyric acid versus *C. sporogenes* relative abundance in cecum. All graphs in panel f-j show median and IQR and statistical analysis was done across groups within each metabolite measured using One-way ANOVA (panel f) or Kruskal Wallis tests (panel g-j), using uncorrected Fisher’s LSD or Dunn’s posthoc tests to compare between individual groups. P values are shown in the figure panels. For panel f-i, n = 5 biologically independent animals per group was used for statistical analysis except ‘Normal Trp+Pectin’ (n = 3) and ‘High Trp’ (n = 4). For panel j, n = 5 biologically independent animals per group was used for statistical analysis, except one value for ILA was excluded as an extreme outlier (Grubbs test, alpha < 0.01). Source data

Having explored the relations between substrate availability and microbial tryptophan metabolism in vitro, we investigated whether substrate availability affected concentrations of circulating tryptophan metabolites in vivo. Four groups of GF mice (*n* = 5 per group) were dosed with our three-species defined community and, after an initial run-in period, fed for 2 weeks either a normal (2 g kg^−1^) diet or a high-tryptophan (16 g kg^−1^) diet, containing either no pectin or 50 g kg^−1^ pectin (Fig. 4a and Extended Data Fig. 5). Tryptophan and pectin supplementation did not affect the water or total food intake, but the calculated tryptophan intake was, as expected, six- to tenfold higher for the high-versus-normal tryptophan (Trp) groups (Extended Data Fig. 5a–c). There was no significant difference in the total caecal and colonic bacterial loads among the groups after 2 weeks of consuming the diets (Extended Data Fig. 5d). However, pectin consumption reduced the relative abundance of *C. sporogenes* in both the caecum and colon, whereas relative abundances of *E. coli* and *B. thetaiotaomicron* were similar between the groups (Fig. 4b and Extended Data Fig. 5e). In agreement with the in vitro results, indole concentrations in the caecum and colon were consistently lower in the presence of pectin in the diet (Fig. 4b,c and Extended Data Fig. 5e–g). Notably, concentrations of indole were not related to the relative abundance of *E. coli* in the individual mice (Fig. 4b and Extended Data Fig. 5e). Normalization of indole concentrations to the abundance of the indole-producing *E. coli* in each animal thus confirmed that the indole-reducing effect of dietary pectin was explained by a reduction of the production of indole from each bacterial cell, rather than by a decrease in the number of producing cells (Fig. 4d and Extended Data Fig. 5f,g). Caecum and colon IPA concentrations were very low (Extended Data Fig. 5h,i), and ILA was below detection level in the intestinal samples, suggesting that these compounds are efficiently absorbed from the gut. In serum, we found higher concentrations of ILA, IAcrA and IPA in mice fed the high-tryptophan diets, confirming that increased tryptophan availability led to increased Stickland fermentation in vivo (Fig. 4e). However, there was no significant difference between the absolute serum concentrations of tryptophan among the groups (Fig. 4e), probably owing to a tight host regulation of circulating free tryptophan concentration in the serum as reported earlier^22,48^. ILA, IAcrA and IPA concentrations in serum did not follow the abundance of *C. sporogenes* in the caecum or colon of individual animals. However, normalization of serum metabolite concentrations to the *C. sporogenes* relative abundance in the caecum and colon suggests that each *C. sporogenes* cell produced significantly more ILA, IAcrA and IPA in the presence of pectin (Fig. 4f and Extended Data Fig. 5j), indicating an upregulation of the tryptophan Stickland fermentation pathway in pectin-fed mice. Importantly, this picture was not seen for Stickland fermentation of other substrates, such as valine and leucine, as we observed a clear correlation of their Stickland reaction products, isovaleric acid and isobutyric acid, with *C. sporogenes* abundance independent of diets (Extended Data Fig. 5k). This confirms that the increased amount of tryptophan Stickland fermentation metabolites was explained not by a general increase in the abundance of the producing strain, but by an increased amount of tryptophan available for bacterial fermentation in animals fed with pectin.

### Fibre inhibits indole production in adult human gut microbiota

**Fig. 5:**
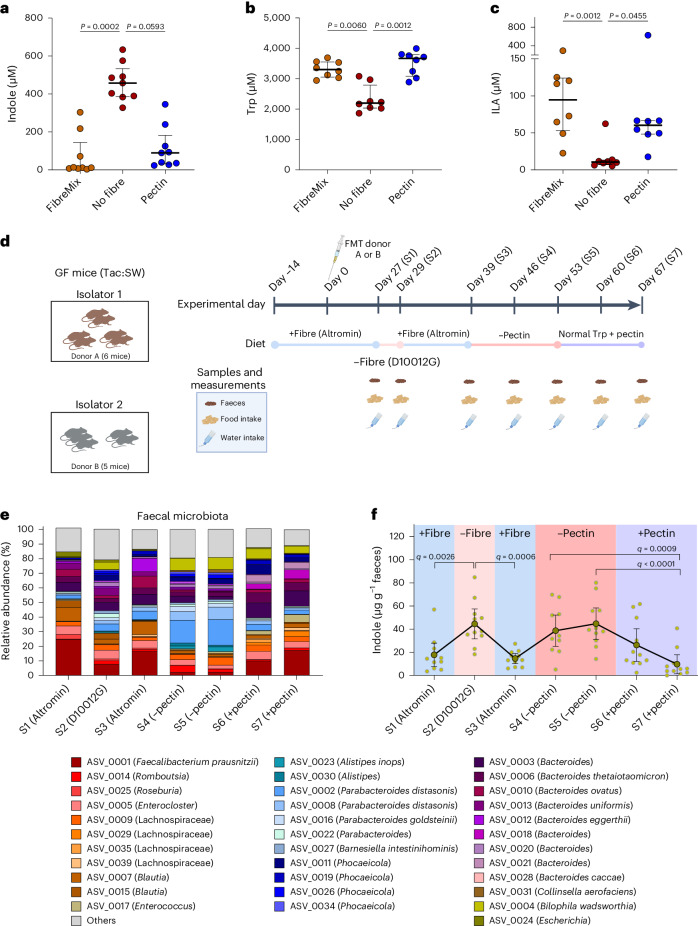
Dietary fibre supplementation inhibits indole production by complex human gut microbial communities both in vitro and in vivo*.* **a**–**c**, Concentrations of tryptophan metabolites indole (**a**) and ILA (**c**) and tryptophan (**b**) in the culture supernatants of nine separate human faecal microbial communities grown in mGAM with no fibre supplementation or supplemented with a mixture of fibres or pectin. Lines and error bars indicate medians and IQRs, respectively. Statistical analysis was done using the Friedman test, with Dunn’s post hoc test (two tailed). *P* values are shown in the figure panels. **d**, Schematic representation of the experimental plan to evaluate the effect of dietary fibre on production of tryptophan metabolites in vivo. GF mice were placed in two groups (*n* = 5 or 6 per group) and fed a complex fibre diet (Altromin 1314) for 14 days before FMT of the mice was done with communities originating from two different human adult donors. Subsequently, the mice remained for another 27 days on the same diet for stabilization. The mice were then fed a diet depleted of fermentable fibres (D10012G) for 2 days before feeding them a complex fibre diet for ten more days. Thereafter, the mice were fed a diet with 2 g kg^−1^ tryptophan without pectin for 2 weeks and then a diet with 2 g kg^−1^ tryptophan and 50 g kg^−1^ pectin for two more weeks. Pectin diet compositions are described in Supplementary Table 1. Faecal samples were collected as shown in the scheme. **e**, The 16S rRNA gene sequencing profiles show the average relative abundance of individual ASVs in faeces across all mice at each sampling point. Only ASVs with relative abundance >5% in at least one sample are shown, and the rest are grouped into ‘others’. **f**, Absolute faecal indole concentrations showing means and 95% CIs, as well as the data point of each individual mouse. Statistical analysis was done using repeated-measure one-way ANOVA, with Sidak’s multiple-comparison test (two tailed; *q* values). The *q* values are shown in the figure panels. Panel **d** was created with BioRender.com. Source data

**Extended Data Fig. 6:**
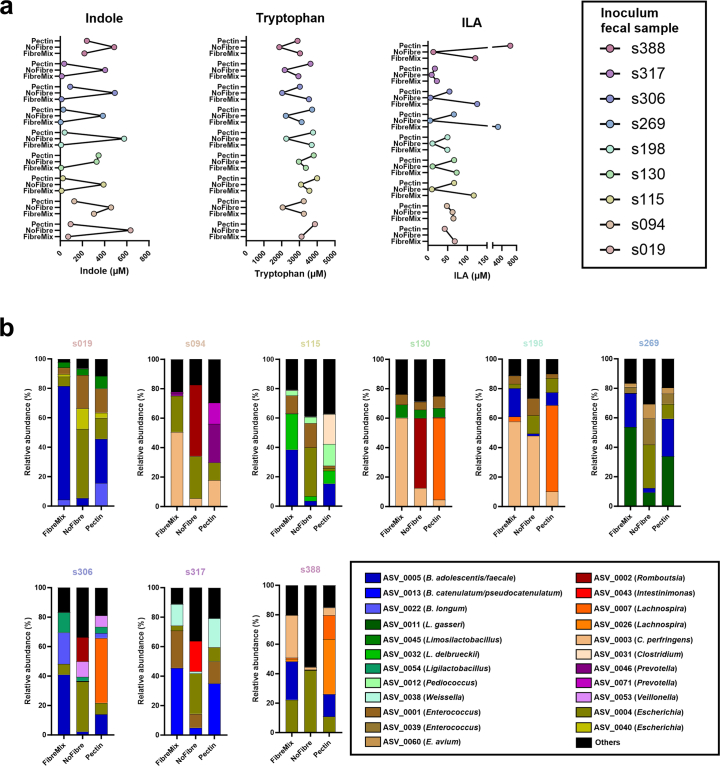
Fibre mixture or pectin supplementation inhibits indole production and stimulates ILA production in complex human gut microbial communities *in vitro.* (**a**) Concentrations of tryptophan metabolites in the individual culture supernatants of nine adult human faecal microbiota in mGAM medium supplemented either with pectin or with the mixture of fibres as described in methods. Results from one sample (S019) is missing for Tryptophan and ILA due to an error with the internal standard in the LC-MS for this sample. (**b**) 16S rRNA gene sequencing profiles show the composition of the microbiota community in each individual cultures. Source data

Given that numerous gut bacterial species, in addition to Enterobacteriaceae, harbour the *tnaA* gene and thus the capacity to produce indole^26^, we next tested whether fibre supplementation inhibits indole production in the complex adult human microbiota. Nine faecal samples obtained from adults were fermented in vitro in mGAM either without supplementation, or supplemented either with pectin or with a mixture of fibres. Indeed, indole production was significantly reduced by the presence of either pectin alone or mixed fibres in the growth medium, whereas high levels of indole were detected in faecal culture supernatants in the absence of fibres (Fig. 5a and Extended Data Fig. 6a). Reduction in indole levels was accompanied by significantly higher tryptophan and ILA concentrations in the presence of fibre mix or pectin (Fig. 5b,c and Extended Data Fig. 6a). IPA was only sporadically detected (measurable in 3 of the 27 fermentations) and, consistently, we did not detect any known IPA producers in the communities (Extended Data Fig. 6b).

**Extended Data Fig. 7:**
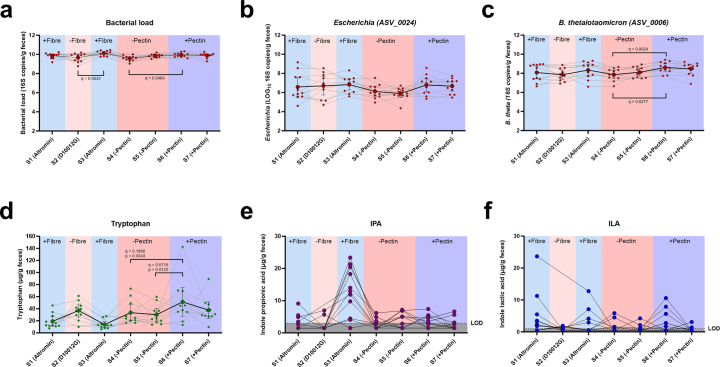
Effect of fibre mixture or pectin supplementation on total bacterial load, *Escherichia* and *B. thetaiotaomicron* as well as concentrations of tryptophan metabolites *in vivo.* (**a**) Total bacterial load in faeces were quantified using qPCR, showing mean and 95% CI, and all samples from individual mice connected with a grey line. (**b**) Absolute abundance of *Escherichia* species (indole producers) across all faecal samples, showing mean and 95% CI, and all samples from individual mice connected with a grey line. (**c**) Absolute abundance of *Bacteroides thetaiotaomicron* (fibre degrader and indole producer) across all faecal samples showing mean and 95% CI and all samples from individual mice connected with a grey line. (**d**) Concentrations of tryptophan across all faecal samples, showing mean and 95% CI. (**e-f**) Concentrations of (**e**) IPA and (**f**) ILA in the faeces of each individual mice at each sampling point, with grey shaded area indicating background noise, and dashed line limit of detection (LOD). For panel a-d statistical analysis (n = 11 biologically independent animals) was done using mixed effects analysis with Sidak’s multiple comparisons tests (q-values) to compare between sampling periods over time. q-values are shown in the figure panels. Due to the limited number of samples above limit of detection statistical test were not perform for data in panel e-f. Source data

Next, we tested the effect of pectin and fibres in vivo using GF mice colonized with two different human microbiota communities derived from adults (Fig. 5d). Mice were initially fed a standard chow diet rich in complex fermentable fibres (Altromin 1314) to facilitate colonization of diverse communities including microbial fibre degraders (Fig. 5d,e). Subsequently, they received a purified diet devoid of fermentable fibres (D10012G) and then went back again on the complex fibre diet (Fig. 5d). These two diets were, however, not matched for nutritional content, and the exact fibre composition is not defined. We therefore subsequently fed the mice with defined purified isocaloric diets containing identical tryptophan levels, with or without pectin, as used in the three-species in vivo study. The mice were thus fed without pectin for 2 weeks and then switched to the diet with pectin for the last 2 weeks (Fig. 5d). Slightly higher bacterial loads were observed when mice were fed with diets containing either complex fibre or pectin, as compared with the fibre-free diets, suggesting that fibre availability supports slightly higher microbial densities in the gut (Extended Data Fig. 7a). However, the relative and absolute abundances of *Escherichia* species remained low and unchanged across the sampling period, while the abundance of *B. thetaiotaomicron* was significantly higher in the faeces of the mice in the pectin feeding versus the no-pectin feeding period (Fig. 5e and Extended Data Fig. 7b,c).

**Fig. 6:**
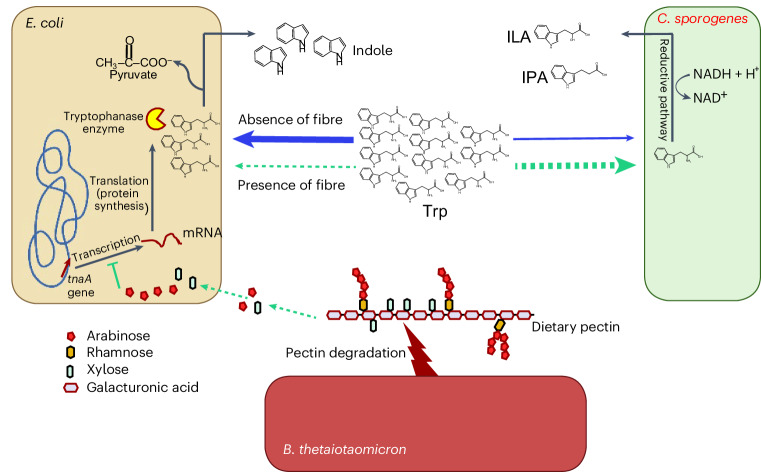
Impacts on tryptophan metabolism mediated by dietary fibre and substrate. In the gut, multiple bacterial species require tryptophan for their metabolism and produce bioactive molecules important for host health. *E. coli* catabolizes tryptophan into indole to generate pyruvate, while *C. sporogenes* regenerates NAD^+^ and produces ILA and IPA through the Stickland fermentation reductive pathway. The fibre degrader *B. thetaiotaomicron* degrades pectin and thereby releases monosaccharides available to *E. coli*. The monosaccharides repress expression of the *E. coli tnaA* gene encoding tryptophanase, thereby making more tryptophan available to Stickland fermenters in the gut environment. Blue arrows show events occurring in the absence of fibre, while green arrows designate events preferentially occurring in the presence of fibre. Thick and thin arrows depict enhanced and reduced flow of tryptophan, respectively. Although *E. coli* is shown here as a representative species of indole producers, we argue that the catabolite repression of the *tnaA* gene is widespread and applies to many other indole producers in the gut. Similarly, in a complex microbiota, fibre degradation and tryptophan utilization can also be performed by other bacterial species than *B. thetaiotaomicron* and *C. sporogenes*, as shown here, thus contributing to the diverse bacterial metabolite accumulation in the gut. Figure was created with BioRender.com.

Indole levels were consistently lower in faeces from mice consuming fibre- or pectin-supplemented diets (sample numbers S1, S3, S6 and S7), irrespective of the community composition and the fibre type used in the diet (Fig. 5f). However, both of the diets without fermentable fibres (sample numbers S2, S4 and S5) promoted production of indole (Fig. 5f). Tryptophan concentrations increased in the periods of pectin feeding versus no pectin feeding; however, this was not statistically significant after correction for multiple testing (Extended Data Fig. 7d). ILA and IPA concentrations were typically higher and/or more often detected in mice fed diets high in fibres; however, they were below detection level in most faecal samples (Extended Data Fig. 7e,f), possibly because of a lack or low abundance of the producer species (Fig. 5e). These results confirm that fibre-mediated inhibition of indole production is not restricted to any particular species or microbial community, but rather a general phenomenon occurring in the human gut microbiota (Fig. 6).

## Discussion

We show that microbial competition for tryptophan determines which tryptophan metabolites are produced by communities of intestinal microorganisms and that this competition is significantly affected by the availability of tryptophan and simple carbohydrates originating from fibre degradation. We propose a model explaining how dietary fibres influence microbiota activity and thereby alter the balance between formation of beneficial (ILA, IPA) and potentially adverse (indole) tryptophan metabolites (Fig. 6). Tryptophan can be converted either into ILA and IPA through Stickland fermentation by microbial species including *C. sporogenes*, or into indole by bacteria expressing the *tnaA*-encoded tryptophanase enzyme, such as *E. coli*. This competition for tryptophan will be influenced by fibre-degrading species such as *B. thetaiotaomicron*, which extracellularly degrades pectin into its constituent monosaccharides. Cross-feeding of monosaccharides to indole-producing species such as *E. coli* leads to catabolite repression, inhibition of *tnaA* gene expression, lower levels of tryptophanase and less tryptophan being converted into indole. Consequently, more tryptophan is available in the gut for other microorganisms to use, leading to increased production of ILA and IPA (Fig. 6).

In agreement with our model, indole production has previously been shown to be inhibited in the presence of starch during batch fermentation using human faecal slurries^49^, and pectin and inulin supplementation has been reported to decrease indole and increase ILA, IAA and IPA accumulation in the medium of a batch culture inoculated with human faeces^50^. In addition, a study on pigs aiming to understand the effect of feeding with non-starch polysaccharides revealed that the intestinal amounts of indole were lower while the amounts of IPA and IAA tended to be higher in pigs fed a high-non-starch polysaccharide diet^51^. The model offers a key to a mechanistic explanation of results obtained in several previous human studies reporting that dietary fibre intake correlates positively to beneficial tryptophan metabolites such as ILA and IPA, but negatively to the potential deleterious metabolites such as IS^10,11,38,51^. High levels of IS are found in the serum of patients with CKD^23^, and increased IS may contribute not only to kidney damage and renal insufficiency but also to atherosclerotic lesions observed in patients undergoing dialysis^52^. Limiting protein intake and increasing dietary fibre intake is reported to reduce serum IS and has been considered as a treatment option for CKD^52–54^. Also, the intestinal pathogen *C. difficile* actively upregulates indole production by indole-producing gut microorganisms that allow *C. difficile* to proliferate and cause persistent infection^20^.

Notably, indole is the most abundant tryptophan metabolite detected in mouse caecal contents as well as in human faeces^21,22^. Thus, downregulation of microbial indole production is expected to contribute considerably to tryptophan availability in the gut.

Our findings provide a rationale for directing gut microbial tryptophan metabolism away from indole production and towards the generation of beneficial Stickland fermentation products including ILA^1–3^, IAcrA^1,4^ and IPA^9,10,13,55^ through dietary fibre interventions with high fibre and/or tryptophan supplementation (Fig. 6). It is worth highlighting that, unlike most previous approaches, this model builds on alteration of microbial activity and gene regulation rather than alteration of microbiota composition and/or abundance of specific producer species. We believe that the future of microbiome research lies in including microbial metabolic activity, not only by assessing the abundance of bacterial genes but also by determining the cues and triggers that regulate their expression. Supporting this belief, a recent analysis of a Dutch-population-based cohort revealed a striking lack of correlation between metagenomics gene abundance and corresponding microbial metabolites^56^. Our finding that interspecies competition for a specific substrate in combination with catabolite repression determines levels of relevant microbial metabolites in the gut can most likely be extrapolated to many other substrates and competition and cross-feeding interactions of the gut microbiome, which remain to be revealed.

## Methods

### Bacterial strains and media

Representative bacterial strains *C. sporogenes* (DSM 795), *P. anaerobius* (DSM 2949), *B. thetaiotaomicron* (DSM 2079) and *E. coli* K12-MG1655 (DSM 18039) were purchased from DSMZ (German Collection of Microorganisms and Cell Cultures). They were revived on mGAM (Nissui, 05426) agar plates; glycerol stocks were prepared and stored in −80 °C until further used. For batch culture experiments, bacterial strains were revived on mGAM agar plates and grown overnight as primary cultures in mGAM broth (Nissui, 05433) under mild shaking conditions. Next morning, they were then diluted again as 0.02 OD_600_ (optical density at 600 nanometer) into 3 ml mGAM broth as secondary cultures and grown for 48 h in mild shaking conditions. Each strain was cultured at least in triplicates. The culture medium without inoculation was used as the control. After 48 h of fermentation, OD_600_ was measured and samples were put on ice. Then, 1 ml each of the samples was centrifuged at 16,000*g* for 10 min at 4 °C, and the supernatants were collected and stored at −20 °C. For supplementation experiments, media were prepared with different amounts of amino acids as indicated in the figures, autoclaved and used as described above. An infant faecal sample was collected and stored in −80 °C as described earlier^3^. For culturing, approximately 50 mg of faeces was inoculated in YCFA medium supplemented with 0.2% each of glucose (Sigma, G8270-1KG), maltose (Merck, 1.05912.0100) and cellobiose (Fluka Analytical, 22150-10G), as described earlier, to support large groups of gut bacterial species^46^, and allowed to grow for 24 h to reach the maximal density. This culture was used as a primary inoculum and diluted 1:100 in YCFA media supplemented with tryptophan (Sigma, T0254) or carbohydrates in the given concentrations. Fermentation was allowed for 24 h; then, pellets and supernatants were collected and stored in −80 °C for further processing.

Adult human faecal samples were cultured in growth medium as described earlier with some modifications^57,58^. For fibre supplementation, the medium was supplemented either with FibreMix (consisting (in g l^−1^) of glucose, 0.4 (Sigma, G8270-1KG); xylan, 0.8 (Sigma, X4252-10G); apple pectin, 0.8 (Sigma, 93854-100G); arabinogalactan, 0.8 (Sigma, 10830-25G); and starch, 5 (Sigma, S9765-100G)) or with apple pectin (7.8 g l^−1^; Sigma, 93854-100G). Collected supernatant samples were processed for metabolite extraction and analysis as described in the next section. All growth experiments were performed inside a Whitley A95 anaerobic workstation maintained at 37 °C, and all the plates or media were incubated inside the workstation at least 24 h before use to maintain anoxic conditions. All biological materials are available either from the authors upon request or from commercial sources.

### Faecal samples

Six infant faecal samples were obtained from the Copenhagen Infant Gut (CIG) cohort established for a separate study with approval from the Data Protection Agency (18/02459) and from the ethical committee^3^. Informed consent was obtained from all parents of infants participating in the CIG study^3^. Eleven faecal samples from healthy adults were obtained from the PRIMA human baseline study (NCT04804319) approved by the Ethical Committee of the Capital Region of Denmark in accordance with the Helsinki Declaration (H-20074067). All participants provided written informed consent.

### Animal experiments

All GF Swiss Webster mice (Tac:SW) used for experiments were bred in the GF facility at the National Food Institute, Technical University of Denmark, maintained on an irradiated chow diet (Altromin 1314, Brogaarden ApS) and transferred to experimental isolators before experiments began. In all experiments, the environment was maintained on a 12 h light and 12 h dark cycle at a constant temperature of 22 ± 1 °C and 55 ± 5% relative humidity, and the air was changed 50 times per hour. For all experiments, the GF status of mice before oral gavage was confirmed by inoculation of faeces from all groups separately into BHI (brain heart infusion) broth (25 °C and 37 °C, aerobic incubation), mGAM broth (37 °C, anaerobic incubation) and plating on blood agar (37 °C, aerobic incubation), and evaluation after 24 h and 2 weeks of incubation. All animal experiments were approved by the Danish Animal Experiment inspectorate (license number 2020-15-0201-00484) and were overseen by the National Food Institute’s in-house Animal Welfare Committee for animal care and use.

#### Mono-colonization experiment

Six GF SW mice (*n*_males_ = 2, *n*_females_ = 4), at the age of approximately 6 weeks, were transferred into an experimental isolator and housed individually (Makrolon type II cage, Techniplast) with bedding, nesting material, a hiding place and a wooden block. Mice had free access to sterile drinking water (Glostrup Hospital) and were maintained on a standard purified diet containing 0.21% tryptophan (D10012G, Research Diets) throughout the experiment. All mice were acclimatized for 7 days before oral gavage with a 200 µl PBS-washed *C. sporogenes* culture (grown overnight in mGAM and washed twice with PBS) and were euthanized after 4 days of colonization. Four female GF SW mice maintained on the same diet for at least 7 days were used as controls for detection of tryptophan catabolites. Mice colonized with *C. sporogenes* and GF control mice were anaesthetized with Hypnorm–midazolam (0.1 ml per 10 g, SC); terminal heart blood (mice colonized with *C. sporogenes*) or portal vein blood (control mice) was collected, and the mice were euthanized by cervical dislocation, before collection of caecum content. Serum was generated from the blood samples after 30 min of coagulation, centrifugation (2,000*g*, 10 min, 4 °C) and aspiration of supernatant into Eppendorf tubes stored at −20 °C until further processing. The caecum content was homogenized 1:4 with sterile Milli-Q water by vortexing and subjected to centrifugation (10,000*g*, 5 min, 4 °C); then, the pellet and supernatant were collected in separate tubes, snap frozen on dry ice and stored at −80 °C until further processing. The primary outcomes assessed in this experiment were the detection and quantity of tryptophan and tryptophan catabolites produced by the Stickland fermentation pathway (IAA, ILA, IAcrA and IPA) in caecum contents and in blood.

#### Three-species community experiment

Twenty GF SW mice (*n*_males_ = 9, *n*_females_ = 11), at the age of approximately 10 weeks, were pseudo randomized into groups based on gender and transferred into 4 separate experimental isolators (each experimental isolator contained a group of 5 mice, including either 3 males and 2 females, or 3 females and 2 females). The mice were housed individually (Makrolon type II cage, Techniplast) with bedding, nesting material, a hiding place and a wooden block. All mice were acclimatized for 7 days before oral gavage with the defined community of bacteria. All mice had free access to sterile drinking water (Glostrup Hospital) and were maintained on an irradiated purified diet named ‘Normal Trp + Pectin’ (A22033102-1.5V, Research Diets) consumed ad libitum from day −7 to day 7. On day 7, all groups except group 1 shifted to diets containing either ‘Normal Trp’ (A18041301R-1.5V, group 2), ‘High Trp + Pectin’ (A22033103-1.5V, group 3) or ‘High Trp’ (A22033101-1.5V, group 4), whereas group 1 continued on ‘Normal Trp + Pectin’ until the experiment ended on day 20 (see all diet compositions in Supplementary Table 1). On day 0, all mice were individually orally gavaged with a 200 µl PBS-washed mixture of individually cultured *B. thetaiotaomicron*, *C. sporogenes* and *E. coli*. These species were cultured individually in mGAM overnight and centrifuged; the cell pellet were washed twice with PBS. Cells of equal OD from all three species were then mixed and prepared for gavage. The 16S rRNA amplicon sequencing of DNA obtained from caecal luminal content confirmed that the mice were colonized only by the three inoculated species. From day 0, fresh faecal samples were obtained every second day and water and food consumption was registered for each individual mouse weekly (day 0, day 7, day 14 and day 20). At day 20, the mice were anaesthetized with Hypnorm–midazolam (0.1 ml per 10 g, SC); terminal heart blood was collected, and the mice were euthanized by cervical dislocation, before collection of gastrointestinal luminal content and tissue. Serum was generated from the heart blood after 30 min of coagulation, centrifugation (2,000*g*, 10 min, 4 °C) and aspiration of supernatant into Eppendorf tubes stored at −20 °C until further processing. Caecum and colon luminal contents were homogenized 1:4 with sterile Milli-Q water by vortexing and subjected to centrifugation (10,000*g*, 5 min, 4 °C); then, the pellet and supernatant were collected in separate tubes, snap frozen on dry ice and stored at −80 °C until further processing. The primary outcomes assessed in this experiment were the detection and quantity of tryptophan catabolites produced by the Stickland fermentation pathway (IAA, ILA, IAcrA and IPA) in the caecum, colon and in serum, and indole in caecum and colon contents.

#### Human faecal microbiota transplantation experiment

Eleven female GF SW mice, at the age of approximately 10 weeks, were pseudo randomized into two groups based on body weight and transferred into two separate experimental isolators. Two or three mice were co-housed together (Makrolon type II cage, Techniplast), with bedding, nesting material, a hiding place and a wooden block. All mice had free access to sterile drinking water (Glostrup Hospital) and were initially maintained on a standard irradiated chow containing a mixture of microbiota-fermentable fibre (Altromin 1314, Brogaarden ApS). All mice were acclimatized for 14 days before faecal microbiota transplantation (FMT) using two separate human adult donors (group 1: 6 mice, donor A; group 2: 5 mice, donor B), at experimental day 0. For FMT, faecal samples were thawed at 4 °C before mixing with three volumes of pre-reduced sterile PBS, vortexing and centrifugation (200*g*, 3 min, 4 °C). Supernatants were further diluted with 1 volume sterile PBS, and 150 µl of this suspension was orally gavaged to each of the mice within 3 h of preparation. After 27 days (S1) of microbiota stabilization on Altromin 1314, all mice were switched to an irradiated purified diet devoid of microbiota-fermentable fibres (D10012G, Research Diets) and then were switched back to the Altromin 1314 diet at day 29 (S2). Subsequently, the mice were switched on day 39 (S3) to the irradiated purified diet ‘Normal Trp’ (A18041301R-1.5V, Research Diets) containing 2 g kg^−1^ tryptophan levels but without pectin, as used in the three-species in vivo study. After 2 weeks on this diet (S4 and S5), the mice were switched on day 53 to the ‘Normal Trp + Pectin’ (A22033102-1.5V, Research Diets) diet containing 2 g kg^−1^ tryptophan with 50 g kg^−1^ pectin for the last 2 weeks of the experiment (S6 and S7), after which the mice were anaesthetized with Hypnorm–midazolam (0.1 ml per 10 g, SC) and euthanized by cervical dislocation. Faecal samples and data on water and food intake were obtained at each of the seven sampling points. The primary outcomes assessed in this experiment were the detection and quantity of tryptophan catabolites in faeces over time.

#### Statistics

All statistical tests for the animal experiments were performed with the GraphPad Prism software (v.10.1.2). Normal distributions were evaluated by the Shapiro–Wilk test. For the three-species defined community experiment, water intake, food intake and estimated tryptophan intake (food intake × Trp content of diets) were compared between groups over time by two-way repeated-measure ANOVA with Bonferroni correction for pairwise comparisons between individual groups. Depending on data distribution, experimental groups were compared using one-way ANOVA or Kruskal–Wallis, with post hoc tests (uncorrected Fisher’s LSD (least significant difference) or uncorrected Dunn’s test) comparing pectin- and no-pectin-feeding groups within the normal and high-Trp-feeding groups and comparing normal and high-Trp-feeding groups within pectin- and no-pectin-feeding groups. For the FMT experiment, faecal concentrations of tryptophan metabolites, bacterial load (qPCR based) and absolute abundance of *Escherichia* and *B. thetaiotaomicron* (16S rRNA sequencing relative abundance × bacterial load) were compared over time across the different sampling periods (S1–S7) by repeated measures one-way ANOVA or mixed-effect analysis (in case of missing samples) with Sidak’s multiple-comparison tests to compare individual sampling points over time. No statistical methods were used to predetermine sample sizes, but our sample sizes are similar to those reported in previous publications^3,12,23^. Data collection and analysis were not performed blind to the conditions of the experiments.

### 16S rRNA gene amplicon sequencing

#### DNA extraction, PCR and library preparation

DNA was extracted from 1 ml faecal culture pellets, approximately 250 mg caecal content, 100 mg colonic content or 50 mg faeces, using the DNeasy PowerLyzer PowerSoil kit (QIAGEN, 12855-100), as described previously^3^, using two to four blank DNA extraction controls. The V3 region of the 16S rRNA gene was PCR amplified using 0.2 µl of Phusion High-Fidelity DNA Polymerase (Thermo Fisher Scientific, F-553L), 4 µl HF (high fidelity) buffer, 0.4 µl dNTP (10 mM of each base), 1 µM forward primer (PBU; 5′-A-adaptor-TCAG-barcode-CCTACGGGAGGCAGCAG-3′) and 1 µM reverse primer (PBR; 5′-trP1-adaptor-ATTACCGCGGCTGCTGG-3′) and extracted DNA diluted to 5 ng µl^−1^ in a 20 µl total reaction volume, with a PCR programme consisting of initial denaturation for 30 s at 98 °C, followed by 30 cycles of 98 °C for 15 s and 72 °C for 30 s, and lastly 72 °C for 5 min to allow final extension before cooling to 4 °C. A total of two no-template controls as well as at least two DNA extraction controls were included per PCR run. The PCR products were purified using the HighPrepTM PCR Magnetic Beads (MAGBIO, AC-60005) with a 96-well magnet stand (MAGBIO, MyMag 96), according to the manufacturer’s recommendations. DNA quantity was measured using Qubit dsDNA HS assay (Invitrogen, Q32851), and samples were pooled to obtain equimolar libraries and sequenced on the Ion S5 System (Thermo Fisher Scientific) using Ion OneTouch 2 with the 520 chip kit-OT2 (Thermo Fisher Scientific, A27751).

#### 16S rRNA gene amplicon sequencing analyses

The 16S rRNA gene amplicon data were processed using our in-house pipeline. In brief, raw amplicon sequences were demultiplexed using Cutadapt (v.4.1)^59^ denoised using DADA2 (v.1.22)^60^ and ASVs (amplicon sequence variant) classified against rdp_train_set_18^61^. Further processing was done using Phyloseq (v.1.42.0)^62^ in R (v.4.2) (R Core Team 2022). For the three-species defined community, in vivo experiment ASVs with less than 100 read counts were removed, and relative abundances were calculated by total sum scaling. The top six ASVs represented on average 99.6% (range, 99.3–99.8%) of all reads in the colon and caecum samples, and were assigned to *Bacteroides* (ASV_2, ASV_4, ASV_5, ASV_6), *Escherichia* (ASV_1) and *Clostridium* sensu stricto (ASV_3). BLAST of the ASV sequences against the 16S rRNA database at NCBI confirmed 100% identity of the three most abundant ASVs towards *B. thetaiotaomicron* (ASV_2), *E. coli* (ASV_1) and *C. sporogenes* (ASV_3), respectively. BLAST against the 16S rRNA database confirmed *Bacteroides* classification of ASV_4, ASV_5 and ASV_6, but no 100% match to any species was obtained. Therefore, these ASVs were additionally searched against the nucleotide collection (GenBank, EMBL, DDBJ, PDB and RefSeq sequences) and were found to match *B. thetaiotaomicron* strain sequences with 100% (ASV_4), 100% (ASV_5) and 98.0% (ASV_6) identity, and these ASVs were collapsed together with ASV_2. The reaming reads represented either ASVs with very low abundance (average relative abundance = 0.39%) matching the same three genus-level taxa as the top six ASVs or sporadically detected ASVs (maximum relative abundance = 0.01%) with high relative abundance in the negative controls (sum of Sphingomonadaceae, *Bradyrhizobium*, *Rhodopseudomonas*, *Brevundimonas*, *Ralstonia*, *Cutibacterium* and *Methylobacterium* on average 86.9%). The relative abundance of ASV_1 thus represented *E. coli*; the combined relative abundances of ASV_2, ASV_4, ASV_5 and ASV_6 represented *B. thetaiotaomicron*; and the relative abundance of ASV_3 represented *C. sporogenes*.

### Quantitative PCR for total bacterial load

As previously described^3^, we quantified the total bacterial load in faeces, caecum and colon samples by qPCR on DNA extracted from these, using universal primers (341F: 5′-CCTACGGGAGGCAGCAG-3′, 518R: 5′-ATTACCGCGGCTGCTGG-3′; final concentration, 0.5 µM each) targeting the V3 region of the 16S rRNA gene. Each reaction was performed in triplicates with 2 µl template DNA, the specified primer concentrations and 2× SYBR Green I Master Mix solution (LightCycler 480 SYBR Green I Master, Roche). Standard curves were generated from known concentrations of tenfold-serial-diluted DNA from *Bifidobacterium longum* subsp. *infantis* DSM 20088. Plates were run on the LightCycler 480 Instrument II with 5 min preincubation at 95 °C, 45 cycles with 15 s at 95 °C, 15 s annealing at 60 °C and 15 s at 72 °C. Data were analysed with the LightCycler 480 Software (v.1.5; Roche).

### Relative gene expression analysis by RT-qPCR

#### RNA extraction

A total of 1 ml of bacterial culture was collected, immediately mixed with two volumes of RNAProtect Bacteria (QIAGEN, catalogue no. 1018380) and pelleted according to the manufacturer’s instructions. The stabilized cell pellets were stored at −80 °C until RNA extraction. RNA was extracted using a combination of enzymatic lysis, bead-beating in hot TRIzol and on-column purification. Briefly, the stabilized pellets were enzymatically lysed for 30 min in a lysozyme solution (15 mg ml^−1^ in TE buffer; L4919 Sigma-Aldrich, Merck) combined with 1:10 (v/v) proteinase K (QIAGEN, catalogue number 19131) treatment. Pellets from early exponential cultures were lysed in a total volume of 220 µl while the 24 h samples were lysed in a total volume of 660 µl split into three separate aliquots of 220 µl each owing to the increased sample material. The lysed cells were then mixed with 1 ml of TRIzol reagent (Invitrogen, catalogue number 15596026) and ~50 mg of glass beads (*Ø* 0.1 mm; QIAGEN, catalogue number 13118-50), incubated at 65 °C for 5 min and beaten for 5 min in a bead beater set at high speed. After the beating, 200 µl of chloroform (VWR Chemicals, 83627.320) was added and the samples were shaken vigorously to mix the phases. Proper phase separation was ensured by centrifugation of the samples at 18,000*g* for 15 min at 4 °C. A total of 700 µl of the resulting RNA-containing aqueous phase was subsequently transferred to a new tube, mixed with 500 µl of ethanol (80% v/v; VWR Chemicals, 20821.310) and spin column purified using an RNeasy Mini Kit (QIAGEN, catalogue number 74104) according to the manufacturer’s instructions. The three aliquots originating from the same 24 h sample were loaded on the same column. During column purification, on-column DNase I (QIAGEN, catalogue number 79254) treatment was included as suggested by the kit manufacturer to remove any trace of genomic DNA. RNA was finally eluted in 50 µl of nuclease-free water. The concentration of the eluted RNA was measured using the QUBIT RNA broad range assay (Invitrogen, Q32853), purity (A260/A280 and A260/A230 ratios) was estimated using a Nanodrop spectrophotometer (Thermo Fisher Scientific) and integrity was investigated by visual inspection using agarose gel electrophoresis (E-Gel EX 1%, Invitrogen, catalogue number G401001). All RNA samples passed the quality control and were stored at −80 °C.

#### cDNA synthesis

cDNA was synthesized from 1,000 ng of RNA using the GoScript Reverse Transcriptase Kit (Promega, A5000) according to the manufacturer’s description with random hexamer primers and a final MgCl_2_ concentration of 5 mM. Identical reactions without the reverse transcriptase were included as negative controls for the qPCR. The cDNA was diluted ten times with nuclease-free water and stored at −20 °C until use.

#### qPCR primer design

The primers used for gene expression analysis are listed in Supplementary Table 2. Nucleotide sequences of the target genes were retrieved from the genome sequences of the organisms. Primers were designed using software from Integrated DNA Technologies. PrimerQuest with standard settings was used to identify potential amplicons and corresponding primer pairs. The primer pairs were then analysed for possible hairpin formation and primer dimer formation using Oligo Analyzer. Primer specificity was ensured using NCBI Primer BLAST^63^, running primer sequences against a custom database composed of the GenBank entries for the genomes of the strains used in the defined community experiments. qPCR test runs (see next paragraph) were conducted to ensure that all primer pairs showed an amplification efficiency above 80% and were free of primer dimer formation and spurious off-target amplification as judged from melting curve analysis.

#### qPCR

qPCR was performed using the intercalating-dye-based GoTaq qPCR master mix kit (Promega, A6001). Briefly, cDNA from an initial RNA input of 10 ng was analysed in a total sample volume of 12 µl with primer concentrations of 800 nM. Samples were mixed in a 384-well PCR plate in technical triplicates. A single replicate of the no-reverse-transcriptase controls and a single replicate of a no-template control were included for all samples and amplicons. Assays were run on a Roche LightCycler 480 qPCR machine using a 40-cycle standard two-step PCR protocol with a combined annealing and amplification step at 60 °C for 1 min. The qPCR protocol was completed by generation of a melting curve.

#### Data analysis and statistics

Melting curve analysis was performed for all assays after their completion to ensure amplification specificity. The raw fluorescence data were analysed using LinRegPCR^64^. This provided a starting concentration of the amplicon (N0) of each qPCR sample (expressed in arbitrary fluorescence units) calculated from the mean amplification efficiency of each amplicon across all samples, the calculated fluorescence threshold and the corresponding quantification cycle^65^. The N0 values were used as the basis for the relative expression analysis. *dnaG*, *gyrA* and *secA* were included as reference genes for all three members of the defined community.

NormFinder^66^ analysis was then performed to select the two reference genes for each individual member that showed the most stable expression level across sample groups. The selected reference genes were then used for normalization to obtain expression ratios for each sample and target gene. Data are presented as the fold change of the expression ratios relative to a reference condition. Unpaired two-tailed *t*-tests were performed on the expression ratios to determine the statistical significance of the relative expression differences. *P* < 0.05 was considered significant.

### Colorimetric indole measurement using Kovacs’ reagent

**Extended Data Fig. 8:**
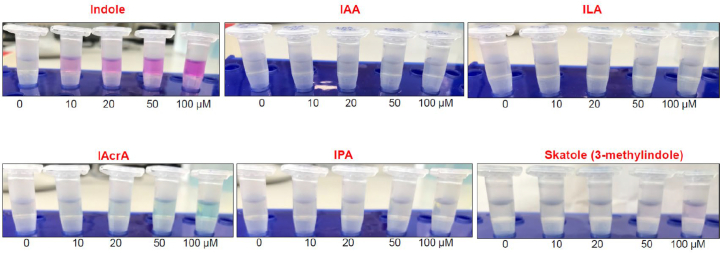
Qualitative analysis of indolic compounds using Kovac’s assay. Kovac’s reagent specificity against 0, 10, 20, 50 and 100 µM concentration of Indole, IAA, ILA, IAcrA, IPA and skatole were assessed and showed that it is highly specific for indole.

The bacterial cultures were centrifuged at 16,000*g* for 10 min at 4 °C, and the supernatant was collected. A total of 250 µl of supernatant was collected in a new 1.5 ml tube, and 250 µl of Kovacs’ reagent (Sigma-Aldrich, Millipore, 109293) was added. The samples were vortexed and incubated at room temperature for 10 min and subjected to a fast spin (approximately 30 s); the top 100–200 µl layer was moved to a 96-well plate, and OD_530 nm_ was measured. Standards (0 µM, 10 µM, 20 µM, 50 µM and 100 µM) of indole (Sigma-Aldrich, 120-72-9), in triplicates, were prepared in the same culture media as that of culture supernatants and processed similar to the samples to generate a calibration curve. Each day, analysis was quantified using the standard curve made on the same day. For quality control, we used six tryptophan metabolites and found that only indole reacts with Kovacs’ reagent (Extended Data Fig. 8).

### Metabolite extraction and profiling

#### Chemicals

Authentic standards of the aromatic amino acids (AAAs) and derivatives were obtained from Sigma-Aldrich, whereas isotope-labelled AAAs used as internal standards (l-phenylalanine (ring-d5, 98%), l-tyrosine (ring-d4, 98%), l-tryptophan (indole-d5, 98%) and IAA (2,2-d2, 96%)) of the highest purity grade available were obtained from Cambridge Isotope Laboratories.

#### Extraction of metabolites from in vitro fermentation samples for AAA metabolite profiling

Culture supernatants from in vitro fermentations were thawed at 4 °C and then centrifuged at 16,000*g* at 4 °C for 10 min. Subsequently, 80 µl was transferred to a new tube and 20 µl internal standard (40 µg ml^−1^) plus 300 µl acetonitrile (Sigma, 1003363276) was added. These samples were vortexed for 10 s and left at −20 °C for 10 min to precipitate the proteins. Then, the samples were centrifuged at 16,000*g*, at 4 °C for 10 min, before 50 µl of supernatant of each sample was diluted with 50 µl of sterile water and transferred to a liquid chromatography vial (equal to a 1:10 dilution of the sample with internal standards having a concentration of 1 µg ml^−1^).

#### Extraction of metabolites from serum samples for AAA metabolite profiling

Serum metabolites were extracted as described earlier^67^. Briefly, sera were thawed at room temperature. Then, 10 µl of internal standard (4 µg ml^−1^) was added to 40 µl of serum. A total of 50 µl of 0.1% formic acid was added into serum, and the mixture was vortexed; then, 400 µl of cold methanol was added and the mixture was vortexed. The samples were then incubated at −20 °C for at least 1 h for protein precipitation. The samples were then centrifuged twice at 16,000*g* at 4 °C for 10 min each to obtain a clear extract, which was then dried under nitrogen gas at 40 °C. The sample was then reconstituted into pure sterile 40 µl Milli-Q water and centrifuged again at 5,000*g* at 4 °C for 5 min to obtain a clear extract and transferred to a liquid chromatography vial for analysis (equal to no dilution of the sample with internal standards having a concentration of 1 µg ml^−1^).

#### Profiling of AAA metabolites from in vitro samples and serum

AAAs and catabolites from in vitro samples and serum were semi-quantified by ultra-performance liquid chromatography mass spectrometry using isotopic internal standards with similar molecular structures as previously published^3^. In brief, the samples (2 µl each) were analysed in random order; however, all samples of the same individual were analysed on the same day with an ultra-performance liquid chromatography quadrupole time-of-flight mass spectrometry system consisting of a Dionex Ultimate 3000 RS liquid chromatograph (Thermo Scientific) coupled to a Bruker maXis time-of-flight mass spectrometer equipped with an electrospray interphase (Bruker Daltonics) operating in positive mode. The analytes were separated on a Poroshell 120 SB-C18 column with a dimension of 2.1 × 100 mm and 2.7 μm particle size (Agilent Technologies) as previously published^3^. Standard mix solutions (0, 0.8 μg ml^−1^, 2 μg ml^−1^ and 4 μg ml^−1^) were analysed as described below. Quality control was ensured by taking standard mix solutions of all the analytes (2 µg ml^−1^) in the culture medium and processing them in a similar manner to the culture supernatant samples to normalize against any loss of the analytes during the processing. In addition, quality control samples and standard mix solutions were analysed before and after all the samples and after every ten samples two standards were analysed, and data were processed using QuantAnalysis v.2.2 (Bruker Daltonics) and a calibration curve (fitted to a quadratic regression) with all standards analysed for each metabolite. The calibration curves were established by plotting the peak area ratios of all of the analytes with respect to the internal standard against the concentrations of the calibration standards.

### Reporting summary

Further information on research design is available in the Nature Portfolio Reporting Summary linked to this article.

### Supplementary information

Supplementary Information Supplementary Tables 1 and 2.

Reporting Summary

### Source data

Source Data Fig. 1 Metabolite source data.

Source Data Fig. 2 Metabolite source data.

Source Data Fig. 3 Metabolite source data and RT–qPCR data.

Source Data Fig. 4 Metabolite source data and relative bacterial abundance.

Source Data Fig. 5 Metabolite source data and relative bacterial abundance.

Source Data Extended Data Fig. 1 Metabolite source data.

Source Data Extended Data Fig. 2 Metabolite source data and relative bacterial abundance.

Source Data Extended Data Fig. 3 Metabolite source data and relative bacterial abundance.

Source Data Extended Data Fig. 4 Metabolites source data and RT–qPCR data.

Source Data Extended Data Fig. 5 Metabolite source data and relative bacterial abundance.

Source Data Extended Data Fig. 6 Metabolite source data and relative bacterial abundance.

Source Data Extended Data Fig. 7 Metabolite source data and relative bacterial abundance.

## Data Availability

All 16S rRNA gene amplicon sequencing data were deposited in the Sequence Read Archive under BioProjects PRJNA1104128 (CIG infant faeces in vitro experiments), PRJNA1102283 (three-species in vivo study), PRJNA1102983 (adult human faecal microbiota in vitro experiment) and PRJNA1102972 (adult human faecal microbiota in vivo experiment). Source data are provided with this paper.

## Code availability

The code used in the study is available via GitHub at https://github.com/MSMortensen/GutMicro_16S_pipeline.
