# Supplementary material for: Dietary fibre directs microbial tryptophan metabolism via metabolic interactions in the gut microbiota
Source: Nat Microbiol. 2024 Jun 25;9(8):1964–78. doi: 10.1038/s41564-024-01737-3 (PMC11306097; doi:10.1038/s41564-024-01737-3)
Supplement: Supplementary file 1 — Supplementary Tables 1 and 2. [file 41564_2024_1737_MOESM1_ESM.pdf]

nature microbiology

Supplementary information

https://doi.org/10.1038/s41564-024-01737-3

Dietary fibre directs microbial tryptophan metabolism via metabolic interactions in the gut microbiota

In the format provided by the authors and unedited

The supplementary data file includes

Supplementary tables

- Supplementary Table 1. Diet formula for *in vivo* experiments
- Supplementary Table 2. List of RT-qPCR primers

Supplementary Table 1-Diet formula for *in vivo* experiments

Ingredients	Diet 1<br>A22033102-1.5V<br>(Normal Trp + Pectin)	Diet 2<br>A18041301R-1.5V<br>(Normal Trp)	Diet 3<br>A22033103-1.5V<br>(High Trp + Pectin)	Diet 4<br>A22033101-1.5V<br>(High Trp)
Total L-amino acids (g)	177.1	177.1	191	191
L-Arginine (g)	*5.9*	*5.9*	*5.9*	*5.9*
L-Histidine-HCl-H_2O (g)	*4.5*	*4.5*	*4.5*	*4.5*
L-Isoleucine (g)	*7.5*	*7.5*	*7.5*	*7.5*
L-Leucine (g)	*15.7*	*15.7*	*15.7*	*15.7*
L-Lysine-HCl (g)	*13.1*	*13.1*	*13.1*	*13.1*
L-Methionine (g)	*5*	*5*	*5*	*5*
L-Phenylalanine (g)	*8.4*	*8.4*	*8.4*	*8.4*
L-Threonine (g)	*7.1*	*7.1*	*7.1*	*7.1*
L-Tryptophan (g)	***2.1***	***2.1***	***16***	***16***
L-Valine (g)	*9.2*	*9.2*	*9.2*	*9.2*
L-Alanine (g)	*5*	*5*	*5*	*5*
L-Asparagine-H_2O (g)	*7*	*7*	*7*	*7*
L-Aspartic acid (g)	*5*	*5*	*5*	*5*
L-Cystine (g)	*4.2*	*4.2*	*4.2*	*4.2*
L-Glutamic acid (g)	*20.7*	*20.7*	*20.7*	*20.7*
L-Glutamine (g)	*17.1*	*17.1*	*17.1*	*17.1*
Glycine (g)	*3*	*3*	*3*	*3*
L-Proline (g)	*17.6*	*17.6*	*17.6*	*17.6*
L-Serine (g)	*9.9*	*9.9*	*9.9*	*9.9*
L-Tyrosine (g)	*9.1*	*9.1*	*9.1*	*9.1*
Corn Starch (g)	372.486	397.486	358.586	383.586
Maltodextrin 10 (g)	132	132	132	132
Sucrose (g)	102.0777	102.0777	102.0777	102.0777
Cellulose (g)	50	50	50	50
**Pectin (g)**	**50**	**0**	**50**	**0**
Soybean Oil (g)	70	70	70	70
t-butylhydroquinone (g)	0.014	0.014	0.014	0.014
Mineral S10022G (g)	0	0	0	0
Mineral S10022C (g)	3.5	3.5	3.5	3.5
Calcium Carbonate (g)	7.34	7.34	7.34	7.34
Potassium Citrate, 1xH_20 (g	2.4773	2.4773	2.4773	2.4773
Potassium Monobasic (g)	6.86	6.86	6.86	6.86
CalciumPhosphate,dibasic (	7	7	7	7
SodiumChloride (g)	2.59	2.59	2.59	2.59
SodiumBicarbonate (g)	7.5	7.5	7.5	7.5
Vitamin Mix V10037 (g)	15	15	15	15
Choline Bitrartrate (g)	2.5	2.5	2.5	2.5
Dye Red (g)	0	0.05	0	0
Dye Yellow (g)	0	0	0.025	0.05
Dye Blue (g)	0.05	0	0.025	0
Total (g)	1008.495	983.495	1008.495	983.495

	Diet 1	Diet 2	Diet 3	Diet 4
Calories (kcal)	3925	3925	3925	3925
Tryptophan (g/kg)	2.08231077	2.135242172	15.86522491	16.26851179
Pectin (g/kg)	49.57882786	0	49.57882786	0
Tryptophan %	0.000535032	0.000535032	0.004076433	0.004076433
Pectin %	0.012738854	0	0.012738854	0

Supplementary Table 2-List of RT-qPCR primers

Name	Sequence (5' - 3')	Gene
Escherichia coli MG1655 (NCBI Reference Sequence: NC_000913.3)		
Ec_tnaA_qPCR-F	GACTGGCTGGCTTATCGTATC	tnaA
Ec_tnaA_qPCR-R	GTTTACCGGCATCAACGAATG	
Ec_araA_qPCR-F1	CATCTCGGTATTGGTGGTAAGG	araA
Ec_araA_qPCR-R1	TGTCGATGCAGTTAACCAGTAG	
Ec_araF_qPCR-F2	ATTGGCTGATCGTCGGTATG	araF
Ec_araF_qPCR-R2	TGCTTTAGACAGTTCGCTCAC	
Ec_rhaA_qPCR-F2	GATGAGGTGATCAGCGAGAAG	rhaA
Ec_rhaA_qPCR-R2	ATTGGAGCCAACCGTGTAG	
Ec_rhaT_qPCR-F2	TACGCTGATGACGCCAATTATC	rhaT
Ec_rhaT_qPCR-R2	GAGTTACAATCCCTACGCCAATC	
Ec_xylA_qPCR-F1	GCTACTGGCACACCTTCTG	xylA
Ec_xylA_qPCR-R1	CTCAAATGCGACATCTGCTTTAC	
Ec_xylG_qPCR-F1	AGGTATCGCCATCATTCATCAG	xylG
Ec_xylG_qPCR-R1	CGTAGCGTCATCAGGTCATAATC	
Ec_dnaG_qPCR-F	GAAGGCTATATGGACGTGGTG	dnaG
Ec_dnaG_qPCR-R	CGCCGTCATAACAGCAAATG	
Ec_gyrA_qPCR-F2	GTGACAAACGTCGTACTGAAATC	gyrA
Ec_gyrA_qPCR-R2	GATACTTAACGTAGCCCTGGTG	
Ec_secA_qPCR-F2	GCTGGTTCTTCCCGTTTCTAC	secA
Ec_secA_qPCR-R2	CCTGGCTTCATACCCAGTTTAC	
Bacteroides thetaiotaomicron DSM 2079 (NCBI Reference Sequence: NC_004663.1)		
Bt_tnaA_qPCR-F	GATGCTGGGTGATGAAAGTTATG	tnaA (BT_RS07555)
Bt_tnaA_qPCR-R	CGTCCTTGATGGGTAGGAATG	
Bt_gyrA_qPCR-F	CGTGGGTGAAGTATTGGGTAAG	gyrA
Bt_gyrA_qPCR-R	GGGCTATCACCATCTACAGAAC	
Bt_dnaG_qPCR-F	CTTCGCCCGCAAACATAATTC	dnaG
Bt_dnaG_qPCR-R	CGTTTGATCGGATCTCTTCCC	
Bt_secA_qPCR-F	CGATGAGGTTGACTCGGTATTG	secA
Bt_secA_qPCR-R	GAGCTTCTACCAGACGTTCTAC	
