# Supplementary material for: Dietary fibre directs microbial tryptophan metabolism via metabolic interactions in the gut microbiota
Source: Nat Microbiol. 2024 Jun 25;9(8):1964–78. doi: 10.1038/s41564-024-01737-3 (PMC11306097; doi:10.1038/s41564-024-01737-3)
Supplement: Supplementary file 2 — Reporting Summary [file 41564_2024_1737_MOESM2_ESM.pdf]

nature portfolio

Corresponding author(s): Tine Rask Licht

Last updated by author(s): Apr 25, 2024

Reporting Summary

Nature Portfolio wishes to improve the reproducibility of the work that we publish. This form provides structure for consistency and transparency in reporting. For further information on Nature Portfolio policies, see our Editorial Policies and the Editorial Policy Checklist.

Statistics

For all statistical analyses, confirm that the following items are present in the figure legend, table legend, main text, or Methods section.

n/a	Confirmed	
☐	☒	The exact sample size (n) for each experimental group/condition, given as a discrete number and unit of measurement
☐	☒	A statement on whether measurements were taken from distinct samples or whether the same sample was measured repeatedly
☐	☒	The statistical test(s) used AND whether they are one- or two-sided<br>*Only common tests should be described solely by name; describe more complex techniques in the Methods section.*
☐	☒	A description of all covariates tested
☐	☒	A description of any assumptions or corrections, such as tests of normality and adjustment for multiple comparisons
☐	☒	A full description of the statistical parameters including central tendency (e.g. means) or other basic estimates (e.g. regression coefficient) AND variation (e.g. standard deviation) or associated estimates of uncertainty (e.g. confidence intervals)
☐	☒	For null hypothesis testing, the test statistic (e.g. F, t, r) with confidence intervals, effect sizes, degrees of freedom and P value noted<br>*Give P values as exact values whenever suitable.*
☒	☐	For Bayesian analysis, information on the choice of priors and Markov chain Monte Carlo settings
☒	☐	For hierarchical and complex designs, identification of the appropriate level for tests and full reporting of outcomes
☐	☒	Estimates of effect sizes (e.g. Cohen's d, Pearson's r), indicating how they were calculated

Our web collection on statistics for biologists contains articles on many of the points above.

Software and code

Policy information about availability of computer code

Data collection	No software was used
Data analysis	Quantification of tryptophan and tryptophan metabolites in samples from in vitro fermentation and from mice cecum and serum were performed using QuantAnalysis version 2.2 (Bruker Daltonics, Bremen, Germany). Gut microbiota data for in vitro infant fecal community and for defined community mice experiments were processed by CLC Genomic Workbench (v8.5. CLCbio, Qiagen, Aarhus, DK), QIIME vl.967, and DADA2 pipeline (v.1.14). Gut microbiota data for in vitro defined community, in vitro adult fecal community and adult fecal mice experiments were processed using our in-house pipeline available on GitHub (https://github.com/MSMortensen/GutMicro_16S_pipeline). qPCR data were processed by the LightCycler® 480 Software v.1.5. Statistical analyses were performed using QIIME (vl.967), R (4.1.0), Microsoft Excel 2016 and GraphPad Prism (v9.5.0).

For manuscripts utilizing custom algorithms or software that are central to the research but not yet described in published literature, software must be made available to editors and reviewers. We strongly encourage code deposition in a community repository (e.g. GitHub). See the Nature Portfolio guidelines for submitting code & software for further information.

Data

Policy information about availability of data

All manuscripts must include a data availability statement. This statement should provide the following information, where applicable:

- Accession codes, unique identifiers, or web links for publicly available datasets
- A description of any restrictions on data availability
- For clinical datasets or third party data, please ensure that the statement adheres to our policy

There is no restrictions on the data. All data supporting the findings of this study are available within the paper and its Supplementary Information. All 16S rRNA gene amplicon sequencing data were deposited in the Sequence Read Archive (SRA) under the BioProjects PRJNA1104128 (CIG infant feces in vitro experiments), PRJNA1102283 (three species in vivo study), PRJNA1102983 (adult human faecal microbiota in vitro experiment) and PRJNA1102972 (adult human faecal microbiota in vivo experiment).

Human research participants

Policy information about studies involving human research participants and Sex and Gender in Research.

Reporting on sex and gender	Sex and gender-analysis were not performed while collecting human fecal samples.
Population characteristics	n/a
Recruitment	n/a
Ethics oversight	n/a

Note that full information on the approval of the study protocol must also be provided in the manuscript.

Field-specific reporting

Please select the one below that is the best fit for your research. If you are not sure, read the appropriate sections before making your selection.

☒ Life sciences ☐ Behavioural & social sciences ☐ Ecological, evolutionary & environmental sciences

For a reference copy of the document with all sections, see nature.com/documents/nr-reporting-summary-flat.pdf

Life sciences study design

All studies must disclose on these points even when the disclosure is negative.

Sample size	No sample-size estimation was performed. For in vitro and vivo study, six infant fecal communities were used from our previous study (PMID: 34675385) and selected based on presence or absence of bacterial species Peptostreptococcus anaerobius and eleven adult fecal communities obtained from PRIMA human baseline study (NCT04804319).
Data exclusions	Due to the loss of one DNA sample during analysis, 0.2 % GMC sample of 23.11-2 in supplementary figure 3 is not reported. Indole concentration from colon of three mice were excluded (supplementary figure 5e) due to the concentrations below detection limit.
Replication	All Experiments were performed with appropriate biological and technical replicates. The number of replication has been described in figure legends or in the methods.
Randomization	n/a, observational study
Blinding	n/a, observational study

Reporting for specific materials, systems and methods

We require information from authors about some types of materials, experimental systems and methods used in many studies. Here, indicate whether each material, system or method listed is relevant to your study. If you are not sure if a list item applies to your research, read the appropriate section before selecting a response.

Materials & experimental systems

n/a	Involved in the study	
☒	☐	Antibodies
☒	☐	Eukaryotic cell lines
☒	☐	Palaeontology and archaeology
☐	☒	Animals and other organisms
☒	☐	Clinical data
☒	☐	Dual use research of concern

Methods

n/a	Involved in the study	
☒	☐	ChIP-seq
☒	☐	Flow cytometry
☒	☐	MRI-based neuroimaging

Animals and other research organisms

Policy information about studies involving animals; ARRIVE guidelines recommended for reporting animal research, and Sex and Gender in Research

Laboratory animals	All germ free (GF) Swiss Webster mice (Tac:SW) used for experiments were bred in the GF facility at the National Food Institute, Technical University of Denmark, maintained on an irradiated chow diet (Altromin 1314, Brogaarden ApS, Lynge, Denmark) and transferred to experimental isolators before experiments began. Mice used for experiments were between 6-10 weeks.
Wild animals	No wild animals were used in the study.
Reporting on sex	Animal sex was not considered as analysis only involved effect of diet on microbial activity and not on the host. However, animals from both male and female were taken for defined community study and pseudo randomized into groups based on gender and transferred into 4 separate experimental isolators (each experimental isolator contained a group of 5 mice, including either 3 males + 2 females or 3 females + 2 females). For adult community study, Eleven female GF SW mice were at the age of approximately 10 weeks pseudo randomized into two groups based on body weight and transferred into two separate experimental isolators. They are described in details in the methods section of the manuscript.
Field-collected samples	No field-collected samples were used in the study.
Ethics oversight	All animal experiments were approved by the Danish Animal Experiment inspectorate (License Number: 2020-15-0201-00484) and were overseen by the National Food Institute's in-house Animal Welfare Committee for animal care and carried out in accordance with existing Danish guidelines for experimental animal welfare.

Note that full information on the approval of the study protocol must also be provided in the manuscript.
